# Temporal and region-specific tau hyperphosphorylation in the medulla and forebrain coincides with development of functional changes in male obese Zucker rats

**DOI:** 10.1152/jn.00409.2023

**Published:** 2024-02-28

**Authors:** Paromita Das-Earl, Derek A. Schreihofer, Nathalie Sumien, Ann M. Schreihofer

**Affiliations:** ^1^Department of Physiology and Anatomy, University of North Texas Health Science Center, Fort Worth, Texas, United States; ^2^Department of Pharmacology and Neuroscience, University of North Texas Health Science Center, Fort Worth, Texas, United States

**Keywords:** Alzheimer’s disease, arterial pressure telemetry, insulin signaling, metabolic syndrome, Morris water maze

## Abstract

Metabolic syndrome (MetS) is associated with development of tauopathies that contribute to cognitive decline. Without functional leptin receptors, male obese Zucker rats (OZRs) develop MetS, and they have increased phosphorylated tau (ptau) with impaired cognitive function. In addition to regulating energy balance, leptin enhances activation of the hippocampus, which is essential for spatial learning and memory. Whether spatial learning and memory are always impaired in OZRs or develop with MetS is unknown. We hypothesized that male OZRs develop MetS traits that promote regional increases in ptau and functional deficits associated with those brain regions. In the medulla and cortex, tau-pSer^199,202^ and tau-pSer^396^ were comparable in juvenile (7–8 wk old) lean Zucker rats (LZRs) and OZRs but increased in 18- to 19-wk-old OZRs. Elevated tau-pSer^396^ was concentrated in the dorsal vagal complex of the medulla, and by this age OZRs had hypertension with increased arterial pressure variability. In the hippocampus, tau-pSer^199,202^ and tau-pSer^396^ were still comparable in 18- to 19-wk-old OZRs and LZRs but elevated in 28- to 29-wk-old OZRs, with emergence of deficits in Morris water maze performance. Comparable escape latencies observed during acquisition in 18- to 19-wk-old OZRs and LZRs were increased in 28- to 29-wk-old OZRs, with greater use of nonspatial search strategies. Increased ptau developed with changes in the insulin/phosphatidylinositol 3-kinase (PI3K)/Akt signaling pathway in the hippocampus and cortex but not medulla, suggesting different underlying mechanisms. These data demonstrate that leptin is not required for spatial learning and memory in male OZRs. Furthermore, early development of MetS-associated autonomic dysfunction by the medulla may be predictive of later hippocampal dysfunction and cognitive impairment.

**NEW & NOTEWORTHY** Male obese Zucker rats (OZRs) lack functional leptin receptors and develop metabolic syndrome (MetS). At 16–19 wk, OZRs are insulin resistant, with increased ptau in dorsal medulla and impaired autonomic regulation of AP. At 28–29 wk OZRs develop increased ptau in hippocampus with deficits in spatial learning and memory. Juvenile OZRs lack elevated ptau and these deficits, demonstrating that leptin is not essential for normal function. Elevated ptau and deficits emerge before the onset of diabetes in insulin-resistant OZRs.

## Graphical abstract

**Figure:**
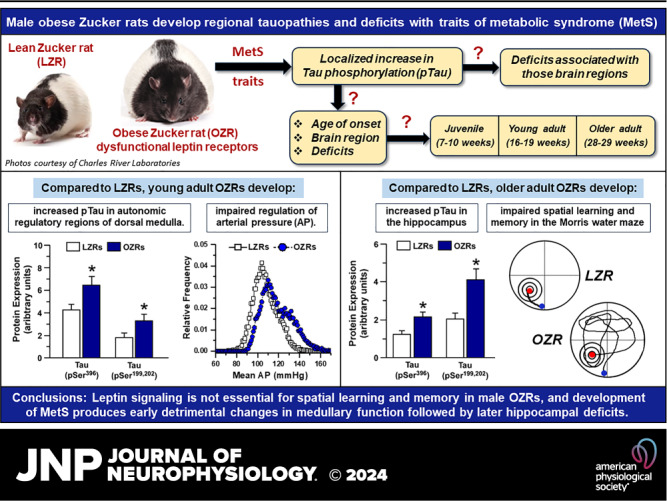

## INTRODUCTION

Excess weight gain with visceral adiposity fosters a cluster of deleterious physiological traits known as metabolic syndrome (MetS) that include insulin resistance, elevated plasma triglycerides with reduced HDL-cholesterol, and hypertension with impaired short-term control of arterial pressure (AP) by baroreflexes (1–3). These traits promote the development of cardiovascular disease and type 2 diabetes mellitus (T2DM) and increase the all-cause risk of premature mortality (2, 4, 5). Physiological traits of MetS are also associated with the development of neurodegenerative disease and cognitive decline (6–8). For example, poorly controlled blood glucose and associated vascular complications of T2DM are established risk factors for Alzheimer’s disease (AD) and related dementias (ADRDs; Refs. 9–11). Because deleterious cognitive consequences brought about by the development of MetS traits are largely irreversible, prevention and intervention with early detection are crucial for reducing the occurrence and progression of ADRDs in the setting of MetS.

The term “type 3 diabetes” describes the strong association between diabetes and Alzheimer’s disease characterized by impaired insulin signaling, glucose utilization, and metabolism in brain regions essential for higher-order functions such as spatial learning and memory (9–13). In studies using humans and animals, elevated circulating insulin and glucose of T2DM are associated with impaired insulin signaling in the brain that causes reduced trafficking of glucose transporters to neuronal membranes and changes in the insulin/phosphatidylinositol 3-kinase (PI3K)/Akt signaling pathway that negatively affect neuronal structure and function (11, 13–15). Interestingly, type 1 diabetes (T1DM), characterized by hyperglycemia with diminished circulating insulin, is also associated with impaired central insulin signaling, neuronal glucose transporter trafficking, and hippocampal glutamate receptor function that likely contribute to observed cognitive deficits (16, 17). Both types of diabetes are associated with central changes in the insulin/PI3K/Akt pathway that alter the phosphorylation of glycogen synthase kinase-3β (GSK3β) to promote excess phosphorylation of the microtubule-associated protein tau (18–21). Hyperphosphorylated tau plays a critical role in the pathology of ADRDs by destabilizing neurons to cause the loss of synapses that disrupts essential connections along with the progressive degeneration of neurons and their associated functions (22–25).

In the hippocampus, GSK3β-mediated hyperphosphorylation of tau impairs synaptic plasticity required for producing long-term potentiation (LTP), which contributes to diminished spatial learning and memory (26–28). The Morris water maze (MWM) is a well-established tool for assessing complex spatial learning and memory that is dependent upon hippocampal LTP, and performance in the MWM is deficient in rats and mice with T1DM and T2DM (29–32). Rats injected with streptozotocin (STZ) to induce T1DM for 10 wk display diminished hippocampal LTP and deficits in the MWM that are prevented with concurrent insulin administration (29). However, in the same study, if insulin treatment begins after 10 wk of STZ-induced T1DM and rats are examined at 20 wk, insulin treatment no longer restores impaired hippocampal LTP or MWM performance. These findings highlight the importance of detecting initial stages of insulin resistance and hyperglycemia to provide effective timely treatments to protect against later irreversible cognitive decline.

Other hormones increased by excess weight gain, such as leptin, appear to have significant roles in neuronal function and morphology, and compromised leptin signaling has been suggested as a key contributor to AD-related pathologies and cognitive impairment (33, 34). Leptin is released from adipocytes and is best known for its roles in metabolism, energy expenditure, and suppression of food intake (35), and the loss or absence of leptin’s actions promotes excess food intake, weight gain, and development of MetS (35–37). Independent of its roles in energy balance and glucose utilization, leptin is neuroprotective and facilitates conversion of short-term potentiation into LTP by enhancing actions of NMDA receptors (34, 38). In a mouse model of AD, central or peripheral treatment with leptin restores diminished phosphorylation of Akt in the hippocampus, reduces neuronal loss, and improves deficiencies in spatial learning and memory (39, 40). As seen with increased circulating insulin in T2DM, excess leptin in the setting of obesity also promotes leptin resistance (35), but whether altered leptin signaling or other traits of MetS foster ADRD-related pathologies is not well understood. In support of the notion that leptin is essential for spatial learning and memory, male obese Zucker rats (OZRs) and *db/db* mice lack functional leptin receptors and have been shown to exhibit impaired hippocampal LTP and performance in the MWM (30, 31, 36, 41, 42). However, other studies using male OZRs report no deficits in hippocampal LTP or performance in the MWM (43), raising the question of whether leptin’s actions are required for the generation of LTP and spatial learning and memory. The use of limited age ranges by many studies leaves open the possibility that these functions could be normal at an earlier age before the onset and progression of potential contributory traits of MetS (30, 31, 42).

This study examined age-matched male OZRs and lean Zucker rats (LZRs) at three age ranges to test the hypothesis that OZRs develop regional rises in phosphorylated tau (ptau) and functional deficits associated with those brain regions along with the onset and progression of MetS traits. We examined juvenile OZRs (7–10 wk), which have excess adiposity with elevated circulating triglycerides, insulin, and corticosterone (44, 45), young adult OZRs (16–19 wk), which also have developed persistent nonfasting hyperglycemia and hypertension with impaired baroreflexes (46, 47), and older adult OZRs (28–29 wk), which also have developed fasting hyperglycemia (48, 49). With these age ranges we examined whether male OZRs develop increased ptau in the medulla with the emergence of brain stem-mediated autonomic dysfunction (46, 50). Although hyperphosphorylated tau has been reported in the medulla of people with AD, this region of the brain has not been examined in rats with MetS. In these leptin receptor-deficient rats, we used the MWM to examine whether deficits in spatial learning and memory develop with MetS traits that increase ptau in the hippocampus. Because multiple regions of the cerebral cortex also affect these functions (51), we determined whether the onset and progression of ptau in the cerebral cortex aligns with changes in the medulla or hippocampus. All rats underwent pretraining for the MWM to focus on hippocampus-dependent spatial learning and memory during the acquisition phase (52–54). We also compared predominant search strategies used by OZRs and LZRs to further characterize spatial learning and memory (54). Finally, we determined whether brain regions with increased ptau also displayed changes in phosphorylated and total Akt and GSK3β protein expression as indications of dysfunctional insulin signaling. This study provides the first examination of whether increased ptau develops in the medulla with the onset of autonomic deficits in the setting of MetS and establishes time courses for development of impaired performance in the MWM and increased ptau in the hippocampus and cerebral cortex in the absence of functional leptin receptors.

## MATERIALS AND METHODS

### Animals

Male OZRs [Lepr (fa/fa)] and LZRs [Lepr (+/+) and (+/fa)] from Charles River Laboratories (Houston, TX) were housed in centralized animal care facilities kept at consistent humidity (60 ± 5%), temperature (24 ± 1°C), and light cycle (lights on 0700–1900 h). Rats had access to water and standard rat chow (Prolab RMH 1800; LabDiet) ad libitum. Rats were not fasted before behavioral testing or euthanasia for tissue collection. The University of North Texas Health Science Center Institutional Animal Care and Use Committee approved all animal experiments, which were performed in accordance with guidelines from the National Institutes of Health’s *Guide for the Care and Use of Laboratory Animals* and the American Physiological Society’s “Guiding Principles for the Care and Use of Vertebrate Animals in Research and Training.” Because female OZRs do not develop several of the pertinent MetS traits in the same age ranges or by the same mechanisms as male OZRs, and their performance in the MWM varies across the estrous cycle, females will be examined in a separate study (55, 56).

### Western Blot Analyses

To determine time courses for changes in ptau expression in major regions of the brain, we collected the intermediate medulla oblongata, hippocampus, and cerebral cortex overlying the hippocampus. Rats were anesthetized with 5% isoflurane in 100% oxygen and rapidly euthanized by decapitation at 7–8 wk, 18–19 wk, or 28–29 wk of age. Brain tissue was dissected on ice, and the hippocampus, cortex, and medulla were rapidly frozen in liquid nitrogen. The tissue was transferred to −80°C until further processing. Frozen brain tissue was powdered in liquid nitrogen and suspended in tissue protein extraction reagent [Thermo Fisher Scientific (TFS)] containing 10 μg/mL HALT protease and phosphatase inhibitor cocktail (TFS). Tissue was homogenized with a handheld homogenizer and centrifuged at 14,000 *g* for 15 min at 4°C to clear the lysate. Lysates were transferred to clean tubes and centrifuged again for 10 min. The supernatant was collected in fresh tubes, and protein concentration of the lysate was measured with a NanoDrop 2000C spectrophotometer (TFS).

Protein lysates (30–40 μg) were mixed in 4× LDS nonreducing sample buffer (TFS) with 10% β-mercaptoethanol and boiled at 95°C for 5 min. Samples were cooled on ice and separated with precast 4–20% minigels (Bio-Rad Laboratories) in a standard Tris-glycine electrophoresis buffer. Separated proteins were transferred onto Amersham Protran nitrocellulose blotting membranes (GE HealthCare Life Sciences) and blocked for 1 h in 5% nonfat milk dissolved in Tris-buffered saline (TBS) containing 0.1% Tween 20 detergent (TBS-Tween). The blots were incubated with their respective primary antibodies (Table 1) overnight at 4°C on an orbital shaker table. Blots were washed 2 × 10 min in TBS-Tween and incubated with horseradish peroxidase (HRP)-conjugated goat anti-rabbit or goat anti-mouse antibodies (1:2,000) for 1–1.5 h. After secondary antibody incubation, blots were washed 2 × 10 min in TBS-Tween, followed by two washes in TBS. Protein bands were visualized with either SuperSignal West Pico PLUS Chemiluminescent Substrate or SuperSignal West Femto maximal sensitivity (TFS). Signal was captured with Alpha Innotech FluorChem software (San Jose, CA) and analyzed with Image J software (NIH). Each blot was stripped with Restore stripping buffer (TFS) and reprobed for β-actin as a loading control.

**Table 1.** Antibodies used for Western blots and immunohistochemistry

Antibody	Species	Application	Dilution	Source
tau-pSer^199,202^	Rabbit polyclonal	Western blot	1:500	Life Technologies Corporation
tau-pSer^396^	Mouse monoclonal	Western blot, IHC	1:200	Life Technologies Corporation
tau 5	Mouse monoclonal	Western blot	1:1,000	Life Technologies Corporation
GSK3β(pSer^9^; D3A4)	Rabbit monoclonal	Western blot	1:500	Cell Signaling Technologies
GSK3β(3D10)	Mouse monoclonal	Western blot	1:500	Cell Signaling Technologies
AKT(pSer^473^; 193H12)	Rabbit monoclonal	Western blot	1:250	Cell Signaling Technologies
pan AKT (40D4)	Mouse monoclonal	Western blot	1:500	Cell Signaling Technologies
β-Actin	Mouse monoclonal	Western blot	1:4,000	Thermo Fisher Scientific

GSK3β, glycogen synthase kinase-3β; IHC, immunohistochemistry.

### Immunohistochemistry

Rats were euthanized by injection of urethane (5 g/5 mL ip) and underwent transcardial perfusion with phosphate-buffered normal saline to clear the blood, followed by perfusion with 4% formaldehyde in sodium phosphate buffer (pH 7.4) for tissue fixation. Brains were postfixed in the formaldehyde for an additional 24–48 h. Then, brains were rinsed in sodium phosphate buffer (pH 7.4) for blocking the brain stem. The medulla oblongata was sectioned in the coronal plane at 30-µm thickness with a vibratome and stored in cryoprotectant solution at −20°C for later immunohistochemistry as previously described (57).

Free-floating sections were stained to localize increased ptau within the medulla. To ensure consistent colorimetric reactions, all staining included brain sections from LZRs and OZRs. Antigen retrieval was performed by incubating the sections in preheated citric acid buffer (10 mM, pH 8.6) at 80°C for 30 min (58). Subsequent incubations were performed with gentle agitation on an orbital shaking table at room temperature unless otherwise specified. Sections were rinsed 3 × 5 min in TBS and blocked for 1 h in TBS cocktail with 10% normal horse serum and 0.3% Triton X-100. Then sections were incubated with tau-pSer^396^ primary antibody (Table 1) for 1 h and transferred to 4°C overnight. The next day, sections were rinsed 3 × 5 min in a TBS cocktail and incubated for 15 min in 1% H_2_O_2_ to quench endogenous peroxidases, followed by incubation with biotinylated donkey anti-rabbit IgG (1:400–800) for 1 h. After rinsing 3 × 5 min in TBS, sections were transferred to an A/B solution (Vectastain Elite ABC kit; Vector Laboratories) to incubate for 45 min. Antigens were revealed by incubation in 3,3′-diaminobenzidine for 2–5 min while sections were monitored for coloration. Stained sections were mounted onto gelatin-coated slides and allowed to dry overnight. Mounted sections were dehydrated by immersion in increasing concentrations of ethanol (70%, 95%, and 100% for 5 min each) and delipidated by immersion in xylenes, and coverslips were affixed with DPX mounting medium (Millipore Sigma).

### Implantation of Transmitters to Record Arterial Pressure by Telemetry

Male LZRs and OZRs aged 14–15 wk were anesthetized with 5% isoflurane in 100% oxygen and maintained with 1.8–2.5% isoflurane. After verification of anesthesia, a vertical midline incision was made in the abdomen to isolate the abdominal aorta. The tip of an HD-S10 transmitter device [Data Science International (DSI)] was inserted into the abdominal aorta caudal to the kidneys and advanced rostrally. The entry site into the vessel was sealed with Vetbond, with blood flow to the hindquarters left intact. The transmitter lead was secured to the abdominal wall with 4-0 Prolene suture. During surgery rats were injected with an analgesic (ketorolac 5 mg/kg im; Hospira Inc., Illinois), and after completion of surgery rats were allowed to regain consciousness and recover in their home cage. Recovering rats were kept warm and monitored until fully conscious and mobile. Each cage was placed on a receiver (PhysioTel model RPC-1; DSI) for continuous measurement of AP by telemetry for several weeks. Recordings of systolic AP, diastolic AP, and mean AP were performed with Ponemah v6.30 software (DSI) and saved as PNMWAV files. Data were extracted from the PNMWAV files and imported into Microsoft Excel for further analysis.

### Morris Water Maze Tests

The Morris water maze (MWM) was used to assess spatial learning and memory. The setup consisted of a 160-cm-diameter steel tub that was 60 cm deep, filled with water maintained at 24 ± 1°C, and made opaque with nontoxic white tempera paint. The testing area was surrounded by external visual cues. All data were recorded with a camera mounted over the pool and analyzed with ANY-maze 5.1 software. The protocol included pretraining, acquisition, retention, and reversal phases. Probe trials were performed before the trials on the last day of the acquisition phase and after the reversal phase trials.

Rats initially underwent 1 day of pretraining with a morning and an afternoon session of three trials/session. They were tasked to swim the narrow path of a straight alley and find a submerged platform in the absence of any external visual cues. This essential pretraining allowed rats to learn the task of turning around and swimming to find a submerged platform and escape the water, which is hippocampus independent, before performing the hippocampus-dependent task of using spatial cues to learn and remember the location of a submerged platform during the acquisition phase (52–54). The next day, rats began a 4-day acquisition phase with three trials/day and a minimum 10-min intertrial interval. The rats were tasked to escape the water by finding a submerged platform in a fixed quadrant, using external visual cues. The rats were released into the pool from three predetermined points and allowed to search for the hidden platform for up to 90 s. The time taken to reach the platform was recorded as the escape latency, and the distance traveled was recorded as path length. Rats unable to locate the platform within 90 s were guided to the platform by gently tapping the platform, and the rats were allowed to remain on the platform for 20 s. Swim speed and percentage of time in the target quadrant were also measured. Before the fourth day’s acquisition trials, a probe test was performed to assess spatial reference memory. The platform was removed, and rats were allowed to swim for 30 s before being removed from the water. Time spent in the target quadrant that previously contained the platform was measured. After 2 days of rest, on *session day 5* rats performed three trials to assess retention of the platform location from the acquisition phase. In the reversal phase on *session days 6* and *7*, the platform was moved to a quadrant opposite to the initial location to assess the ability to extinguish previous spatial learning and learn a new location for the platform. A second probe test was performed after the reversal phase trials were completed to assess their ability to remember the new location of the submerged platform.

For the characterization of search strategy patterns in the MWM tests in LZRs and OZRs, path maps obtained from ANY-maze 5.1 software (Stoelting) were assigned a score based on the predominant search strategy employed during each trial (54, 59, 60). If a rat used more than one search strategy during the trial, the predominant strategy was assigned. These categories were evaluated by visual inspection of path maps independently by two investigators blinded to the groups without the use of automated software or algorithms. Swim paths were categorized as thigmotaxis (an indication of anxiety-related behavior), nonspatial search (random search, scanning, or chaining), or spatial search (self-orienting, indirect swim, or direct swim), as previously described (54, 59, 60). The primary goal was to determine whether LZRs implemented spatial search strategies more often than OZRs during the acquisition and retention phases. Incidence of predominant search strategies was displayed as a percentage of trials to account for small differences in group sizes.

### Data Analyses

The mean AP for each rat was derived from 24 consecutive hourly averages that were derived from twelve 20-s averages every 5 min. Standard deviation of the mean AP for each rat was calculated from the 24 consecutive hourly averages to serve as an estimate of within-subject variability of AP (61). To determine whether increased mean AP was indicative of systolic hypertension in OZRs, the number of systolic AP readings of 140–149 mmHg, 150–159 mmHg, or 160–169 mmHg was counted from a week of recordings, using the 5-min values from each rat (62). The within-subject number of systolic events per week in OZRs and LZRs was compared by chi-square analysis to determine overall difference by rat phenotype. Then, pairwise comparisons between OZRs and LZRs for each range of mean AP were made by Mann–Whitney *U* test and corrected for multiple comparisons with the Holm–Sidak method. The 7 days of recordings were also used to construct a frequency distribution of mean AP (∼2,000 values/rat) for the LZRs and OZRs. To control for the different group sizes and occasional absent readings, the frequencies were converted to relative frequencies by dividing the frequency of each mean AP value by the total number of AP values included.

For Western blot analysis, the band density for each protein was measured with ImageJ software (NIH) and normalized to the band density of β-actin. All single-value comparisons between age-matched OZRs and LZRs were performed with unpaired *t* tests. For the MWM tests, the escape latencies, path lengths, percent time spent in target quadrant, and swim speeds were compared by two-way repeated-measures (RM) ANOVA followed by Tukey’s post hoc tests when a significant *F* value was obtained (SigmaPlot 10). The acquisition and retention phases on *days 1–5* were analyzed separately from the reversal phase on *days 6* and *7*. Single-point values during individual probe trials were compared by unpaired *t* tests. Qualitative estimations for incidence of predominant search strategies from all trials performed on *days 1–5* were analyzed with generalized estimating equations (GEEs) and Wald chi-square tests for within-group and between-group comparisons with SPSS version 8 software (54). A value of *P* < 0.05 was considered significant for all statistical analyses, and all *P* values < 0.001 were designated as *P* < 0.001. Graphs and figures were created with Origin 2017, Microsoft Excel 2020, and Canvas X Draw version 20.

## RESULTS

### Young Adult Male OZRs Develop Increased ptau Expression in Medulla and Cerebral Cortex with Deficits in Autonomic Regulation of AP and Nonspatial Learning

#### Increased ptau in medulla and cerebral cortex but not hippocampus of young adult OZRs.

**Figure 1.**
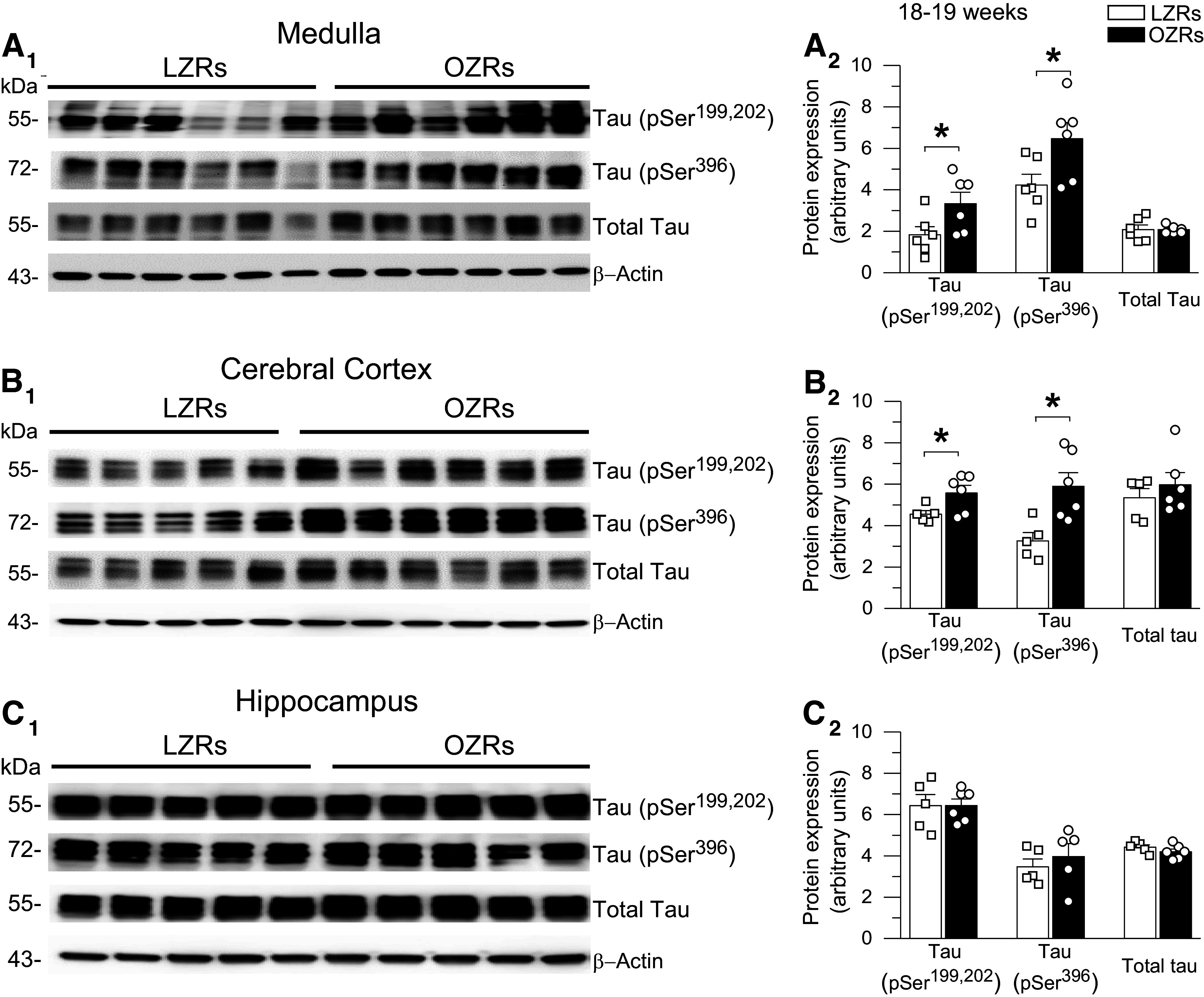
Protein expression of phosphorylated tau is increased in the medulla and cortex but not the hippocampus in young adult obese Zucker rats (OZRs). *A_1_*, *B_1_*, and *C_1_*: immunoblots used to quantify protein expression of tau-pSer^199,202^, tau-pSer^396^, and total tau from intermediate medulla, cerebral cortex, and hippocampus of 18- to 19-wk-old OZRs and lean Zucker rats (LZRs). Band densities were quantified by ImageJ and normalized to band density of β-actin. In the medulla (*A_2_*) and cortex (*B_2_*) expressions of tau-pSer^199,202^ and tau-pSer^396^ were higher in OZRs vs. LZRs, with no differences in total tau. In the hippocampus (*C_2_*) expressions of tau-pSer^199,202^, tau-pSer^396^, and total tau were comparable in OZRs and LZRs. Data are shown as means ± SE. Group sizes were 5 or 6 rats for all comparisons. *P < 0.05, unpaired *t* tests.

**Figure 2.**
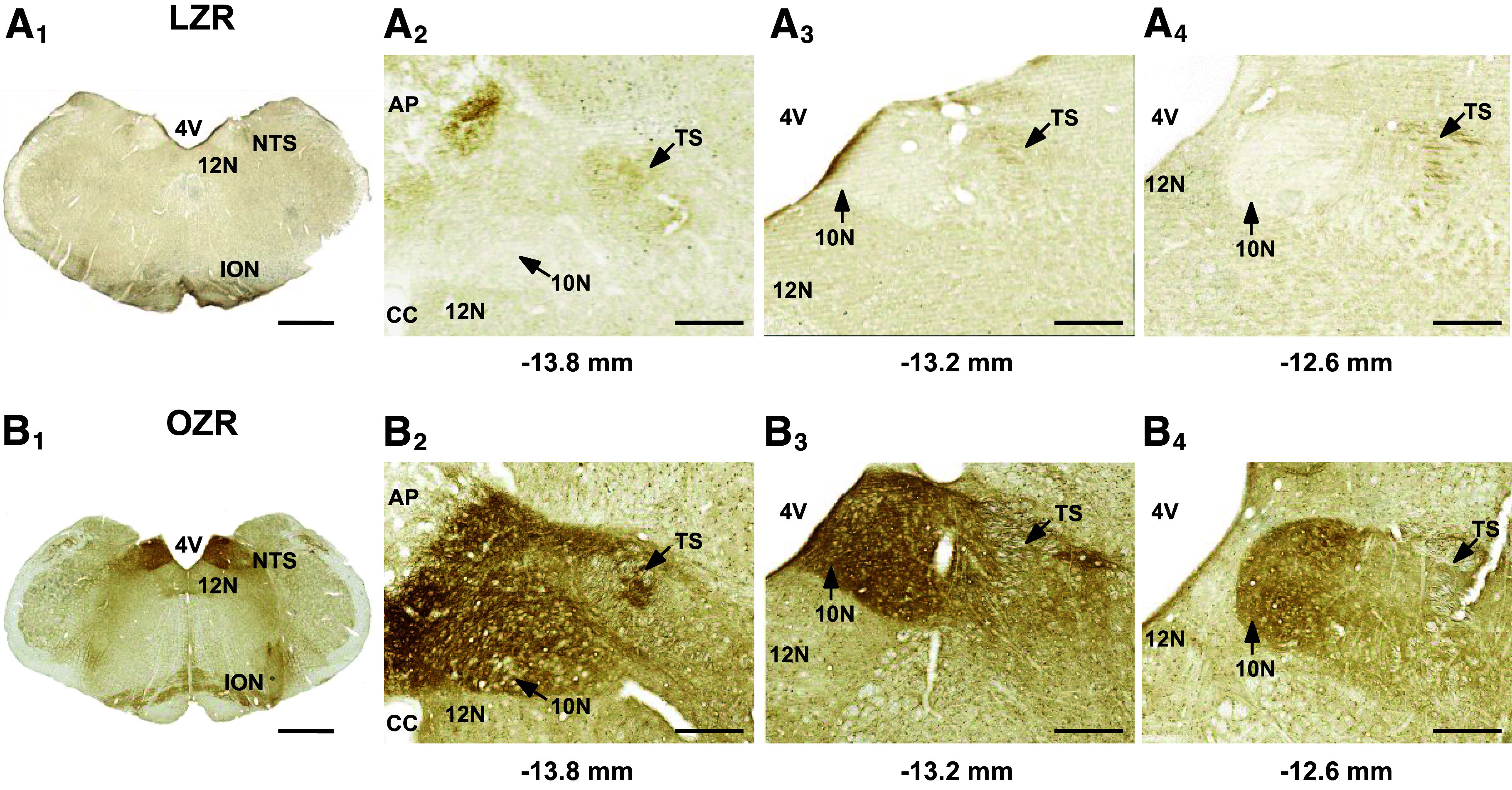
Increased tau-pSer^396^ is concentrated in the dorsal vagal complex in the medulla of a young adult obese Zucker rat (OZR). *A_1_* and *B_1_*: representative photomicrographs (×5 magnification) showing tau-pSer^396^ immunoreactivity in coronal sections of intermediate medulla (bregma −13.2 mm) of an 18-wk-old lean Zucker rat (LZR) (*A_1_*) and an OZR (*B_1_*). In the OZR (120 days old), immunoreactive staining for tau-pSer^396^ was most pronounced in the nucleus tractus solitarii (NTS), dorsal motor nucleus of the vagus (DMNV), and the inferior olivary nucleus (ION). In the LZR (119 days old), no regions of the medulla had the intense staining observed in the OZR. Higher magnification (×20) of the dorsal vagal complex shows intense staining throughout the NTS and DMNV of the OZR (*B_2_*–*B_4_*) that is absent in the LZR (*A_2_–A_4_*) at −13.8 mm, −13.2, and −12.6 mm caudal to bregma. AP, area postrema; CC, central canal; TS, tractus solitarius; 4V, fourth ventricle; 10N, dorsal vagal motor nucleus, 12N, hypoglossal nucleus. Scale bars, 1 mm in *A_1_* and *B_1_* and 200 µm in *A_2_–A_4_* and *B_2_–B_4_*.

At 18–19 wk of age the OZRs weighed ∼222 g more than the LZRs (686.2 ± 29 vs. 464.7 ± 12 g, *n* = 6/group, *P* < 0.001, unpaired *t* test). In the medulla and cerebral cortex, expressions of tau-pSer^199,202^ and tau-pSer^396^ were higher in OZRs compared to LZRs, with no significant difference in total tau protein expression (Fig. 1, *A* and *B*). In contrast, the expression of ptau at these residues in the hippocampus was not different between OZRs and LZRs. (Fig. 1*C*). In the medulla, immunohistochemistry for tau-pSer^396^ revealed intense staining in the OZR but not the LZR that was localized to the dorsal medulla within the nucleus tractus solitarii (NTS) and underlying dorsal motor nucleus of the vagus (DMNV; Fig. 2), which receive visceral autonomic sensory inputs and give rise to parasympathetic innervation of visceral organs, respectively (63, 64). This intense staining was present at all rostro-caudal levels of the dorsal vagal complex examined (Fig. 2, *B2–B4*), in agreement with previously reported changes in NTS and DMNV activation and autonomic control of cardiovascular and gastrointestinal functions in obese and diabetic rats (50, 63–65). In the ventral medulla of the OZR, intense staining for tau-pSer^396^ was present in the inferior olivary nucleus, a region critical for cerebellar function and motor learning (Fig. 2*B1*).

To determine whether elevated ptau expression observed in young adult OZRs was present at an earlier age, we examined 7- to 8-wk-old OZRs and LZRs. These juvenile OZRs weighed ∼81 g more than the LZRs (261.4 ± 6.1 vs. 180.2 ± 6.0 g, *n* = 5/group, *P* < 0.001, unpaired *t* test), but nonfasting blood glucose was comparable (132.1 ± 2.7 mg/dL in OZRs vs. 129.3 ± 2.3 in LZRs, *P* = 0.48, unpaired *t* test). Expression levels of tau-pSer^199,202^ and tau-pSer^396^ and total tau within the medulla and cerebral cortex were not different between these OZRs and LZRs (Supplemental Fig. S1). Together, these data show emergence of elevated expression of tau-pSer^199,202^ and tau-pSer^396^ in the medulla and cerebral cortex in young adult OZRs.

#### Increased AP and variability of AP develop in young adult OZRs.

**Figure 3.**
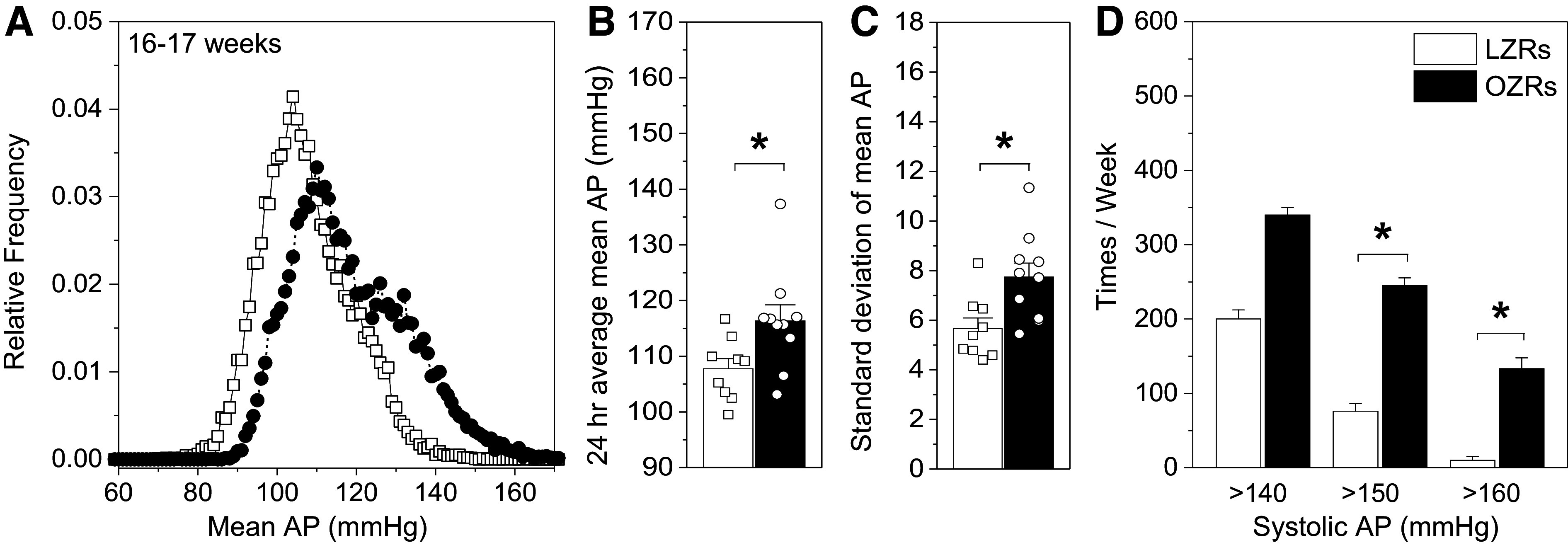
Mean arterial pressure (AP) and variability of AP are elevated in young adult male obese Zucker rats (OZRs) compared with age-matched lean Zucker rats (LZRs). *A*: the relative frequency distribution of mean AP was shifted rightward to higher AP values with a lower peak and wider distribution in OZRs (*n* = 10) vs. LZRs (*n* = 9). Mean AP values were collected in 20-s samples every 5 min over 7 consecutive days and divided by the total number of values (17,553 in LZRs and 19,405 in OZRs). The 24-h mean AP (*B*) and standard deviation of mean AP (*C*) were higher in OZRs vs. LZRs. Values were derived from 24 consecutive hours at 5-min intervals. *P < 0.05, unpaired *t* test. *D: c*ounts of systolic AP readings per week > 140 mmHg showed more frequent systolic events in the 150–159 mmHg and 160–169 mmHg ranges in OZRs vs. LZRs. Chi-square analysis showed a significant effect for phenotype (χ^2^ = 532.89, *P* < 0.001). Pairwise comparisons for each range of systolic AP were made with the Mann–Whitney *U* test corrected for multiple comparisons with the Holm–Sidak method (*P* = 0.008 for each threshold value). *P < 0.05, OZRs vs. LZRs within that range of systolic AP. Data in *B* and *C* are shown as means ± SE.

Continuous recordings of AP by telemetry in conscious, undisturbed, and freely moving young adult OZRs (*n* = 10) and LZRs (*n* = 9) at 16–17 wk of age confirmed modestly elevated mean AP and increased variability of AP in the OZRs (46, 47, 50, 62, 66). Compared with the LZRs, the relative frequency distribution of 24-h mean AP for the OZRs was shifted rightward, with a lower peak and wider distribution (Fig. 3*A*). These changes were reflected in a higher average 24-h mean AP with higher frequencies of systolic AP >150 mmHg and >160 mmHg in the OZRs (Fig. 3, *B* and *D*). In addition, the OZRs had a higher standard deviation of mean AP (Fig. 3*C*), consistent with previous reports of impaired baroreflexes due to diminished activation of the NTS by baroreceptor afferent nerves in young adult male OZRs (46, 47, 50, 66, 67).

#### Development of nonspatial learning deficits without impairment in spatial learning and memory in young adult OZRs.

At the onset of testing with the MWM, the 18- to 19-wk-old OZRs weighed ∼248 g more than the age-matched LZRs (655.0 ± 9.2 vs. 407.3 ± 8.2 g, *n* = 12/group, *P* < 0.001, unpaired *t* test). On the pretraining day, young adult OZRs had longer escape latencies compared with age-matched LZRs during the three trials of the morning session (Supplemental Fig. S2). In contrast, these OZRs significantly improved in the afternoon session, and their escape latencies became comparable to LZRs, suggesting no persistent sensorimotor deficits (Supplemental Fig. S2).

**Figure 4.**
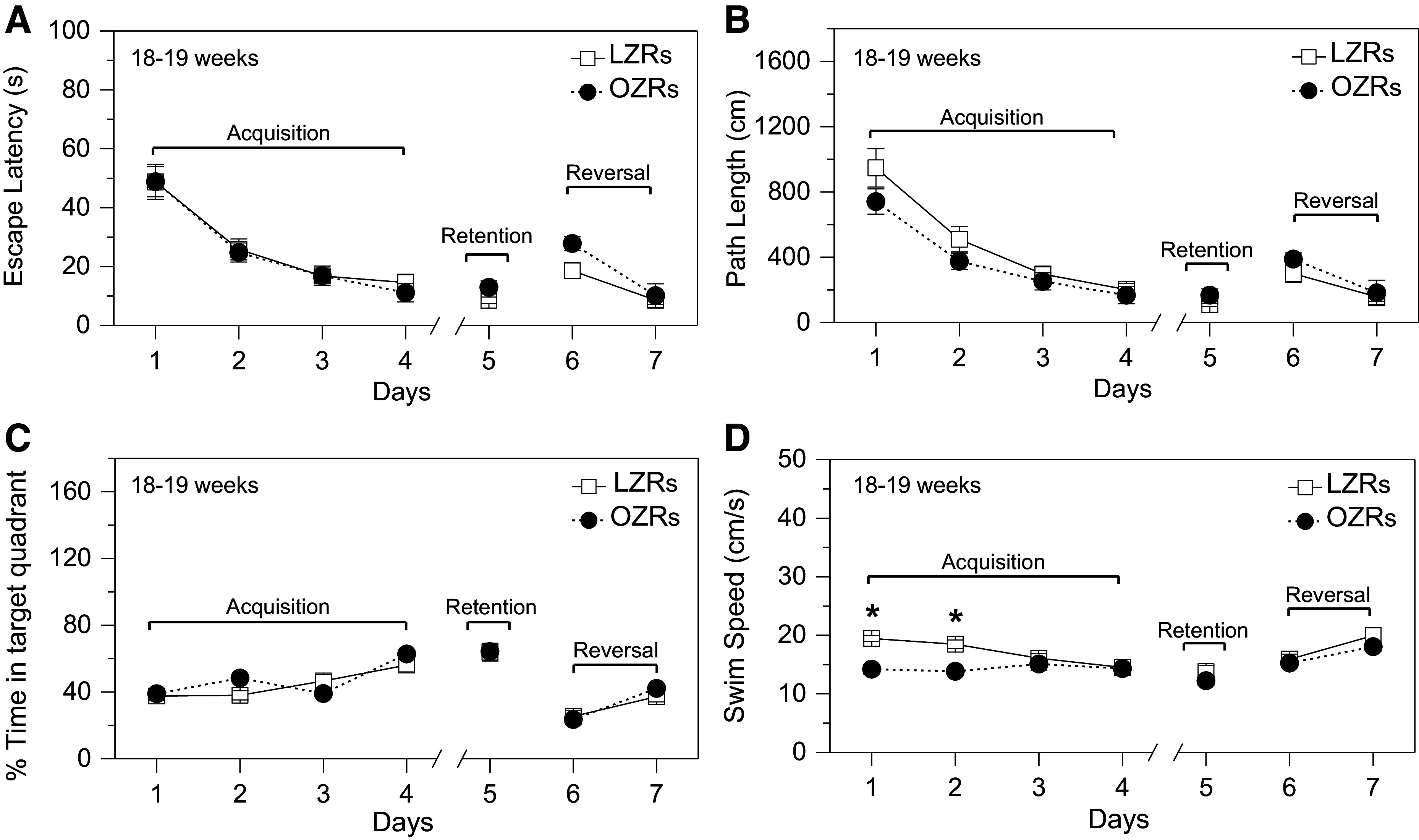
Spatial learning and memory assessed with the Morris water maze (MWM) were comparable in young adult obese Zucker rats (OZRs) and lean Zucker rats (LZRs). Rats were 125 days old on the first day of pretraining for the MWM (*n* = 12/group for all comparisons). *A*: escape latencies decreased with days but were comparable between OZRs and LZRs. See Supplemental Fig. S2 for the pretraining escape latencies for these OZRs and LZRs. *B:* daily path lengths traversed during the 90-s trials decreased with days but were comparable between OZRs and LZRs. *C:* percent time spent in the target quadrant increased with days but was comparable between OZRs and LZRs. *D:* swim speed decreased with days and was slower in OZRs vs. LZRs on *days 1* and *2*. Data are shown as means ± SE. *P < 0.05, OZRs vs. LZRs on that day. *Days 1–5* [*period 1* (P1): acquisition and retention] and *days 6* and *7* [*period 2* (P2): reversal] were analyzed separately by 2-way repeated-measures (RM) ANOVA for days and phenotype, followed by Tukey’s post hoc tests to compare OZRs and LZRs by day when a significant main effect of phenotype was observed. *A*: escape latency: days (P1: *F* = 46.39, *P* < 0.001 and P2: *F* = 26.85, *P* < 0.001) and phenotype (P1: *F* = 1.83E–05, *P* = 0.997 and P2: *F* = 3.37, *P* = 0.080). *B*: path length: days (P1: *F* = 48.89, *P* < 0.001 and P2: *F* = 13.18, *P* = 0.001) and phenotype (P1: *F* = 2.21, *P* = 0.151 and P2: *F* = 0.97, *P* = 0.335). *C*: percent time in target quadrant: days (P1: *F* = 16.84, *P* < 0.001 and P2: *F* = 83.86, *P* < 0.001) and phenotype (P1: *F* = 0.82, *P* = 0.376 and P2: *F* = 0.20, *P* = 0.660). *D*: swim speed: days (P1: *F* = 9.23, *P* < 0.001 and P2: *F* = 23.80, *P* < 0.001) and phenotype (P1: *F* = 7.81, *P* = 0.011 and P2: *F* = 1.16, *P* = 0.292).

**Figure 5.**
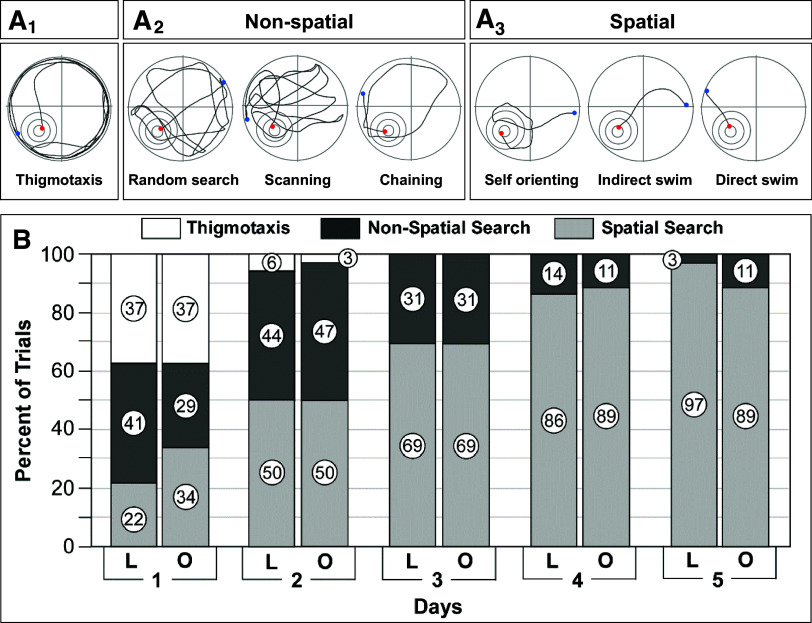
Use of spatial search strategies during the acquisition and retention phases of the Morris water maze (MWM) was comparable in young adult obese Zucker rats (OZRs) and lean Zucker rats (LZRs). *A*: representative examples of swim paths with strategies categorized as thigmotaxis (*A_1_*), nonspatial search (*A_2_*), and spatial search (*A_3_*) (53). *B*: incidence of predominant search strategies for all trials on *days 1–5* displayed as % of total trials in LZRs (L) and OZRs (O) from Fig. 4. Thigmotaxis was present on *days 1* and *2* but absent on *days 3–5* in OZRs and LZRs. The use of spatial search strategies increased with days in OZRs and LZRs as the use of nonspatial search strategies decreased. The data were analyzed by generalized estimating equations (GEEs) and Wald chi-square (54), which showed a significant effect for days (χ^2^ = 883.665, *P* < 0.001) but not phenotype (χ^2^ = 0.133, *P* = 0.715).

During the acquisition phase, escape latencies and path lengths decreased over the 4-day testing period, with no differences between LZRs and OZRs on any of the days (Fig. 4, *A* and *B*). The percentage of time spent in the target quadrant was also comparable in OZRs and LZRs (Fig. 4*C*). On *days 1* and *2*, swim speed in OZRs was slower than that in LZRs (Fig. 4*D*), as previously reported in OZRs (43), with no differences in swim speed in the following days of testing. In the retention phase on *session day 5*, no differences between OZRs and LZRs were observed for escape latency, path length, percent time in the target quadrant, or swim speed (Fig. 4). Examples of swim path categories are depicted in Fig. 5*A*. Analysis of search strategies during the acquisition and retention phases showed a progressive increase in the use of spatial search strategies over the course of 5 days (Fig. 5*B*), as previously reported for the MWM (54). No differences were observed between OZRs and LZRs in their preference for use of spatial versus nonspatial search strategies (Fig. 5*B*), suggesting intact functional telencephalic connections in young adult OZRs (51). Thigmotaxis was observed on *days 1* and *2* in some LZRs and OZRs but absent on *days 3–5*, suggesting reduced anxious behavior with more familiarity performing the tasks.

For the reversal phase on *days 6* and *7*, the escape latency, path length, percent time in the target quadrant, and swim speed were equivalent in LZRs and OZRs on both days (Fig. 4, *A–D*). A probe test performed on *day 4* showed no differences between LZRs and OZRs in the percentage of time spent in the target quadrant (44.7 ± 3.6% vs. 44.5 ± 4.0%) or the annulus around the target at 40 cm (29.5 ± 3.2% vs. 27.5 ± 3.3%) or 20 cm (17.2 ± 2.2% vs. 13.5 ± 2.1%), indicating intact functional reference memory in LZRs and OZRs during acquisition. After reversal phase testing, a second probe test showed no differences between LZRs and OZRs in the percent time spent in the target quadrant (42.4 ± 3.9% vs. 44.2 ± 3.1%) or percent time in the annulus around the target at 40 cm (35.7 ± 3.4% vs. 35.5 ± 3.5%) or 20 cm (26.3 ± 3.2% vs. 19.9 ± 2.7%). These data suggest similar competencies between young adult male LZRs and OZRs in remembering a new location for the platform, which relies on cognitive flexibility using multiple connected regions of the brain (68–71).

We examined 9- to 10-wk-old OZRs (*n* = 6) and LZRs (*n* = 5) to determine whether longer pretraining escape latencies were present in juvenile OZRs. The OZRs weighed ∼162 g more than the LZRs (473.5 ± 14.3 vs. 311.4 ± 7.3 g, *P* < 0.001, unpaired *t* test). During pretraining, these juvenile OZRs and LZRs showed no differences in escape latencies in the morning or afternoon sessions, suggesting that this deficit in nonspatial learning develops in young adult OZRs (Supplemental Fig. S2). Spatial learning and memory were also examined in juvenile OZRs (*n* = 12) and LZRs (*n* = 11) to determine whether they showed any deficits in the acquisition phase of the MWM as previously reported (31). The OZRs weighed ∼156 g more than the LZRs (448.2 ± 10.5 vs. 292.1 ± 6.9 g, *P* < 001, unpaired *t* test). In contrast to previous findings of differences in juvenile OZRs and LZRs (31), no differences were observed for any of the measures during the acquisition, retention, or reversal phases in juvenile OZRs compared with age-matched LZRs (Supplemental Fig. S3).

### Older Adult Male OZRs Develop Increased ptau Expression in the Hippocampus with Deficits in Spatial Learning and Memory

#### Increased ptau in hippocampus and reduced ptau in medulla and cortex in older adult OZRs.

**Figure 6.**
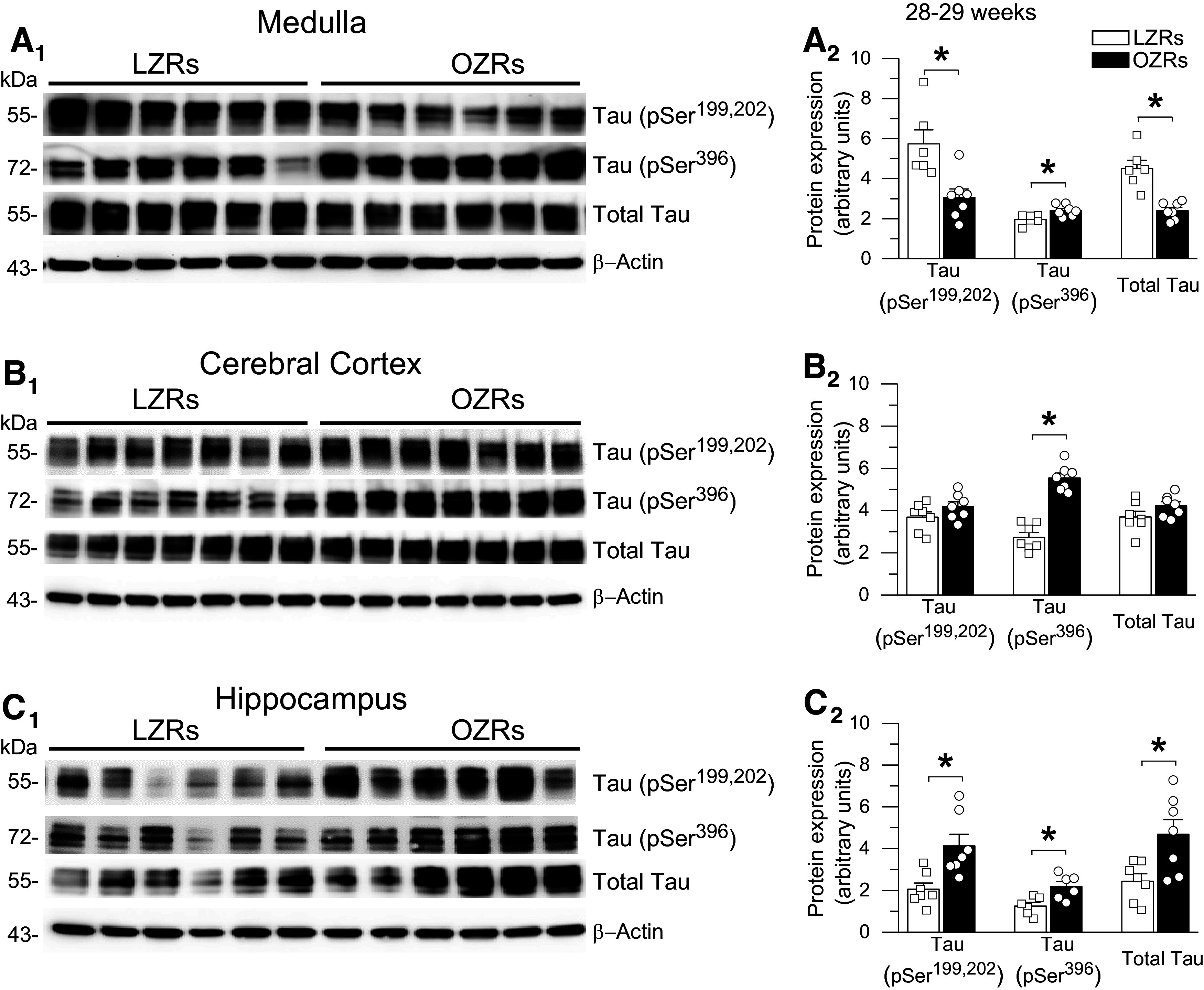
Emergence of increased tau-pSer^199,202^ and tau-pSer^396^ expression in the hippocampus in older adult obese Zucker rats (OZRs). *A_1_*, *B_1_*, and *C_1_*: immunoblots used to quantify protein expression of tau-pSer^199,202^, tau-pSer^396^, and total tau from intermediate medulla (*A_1_*), cerebral cortex (*B_1_*), and hippocampus (*C_1_*) of 28- to 29-wk-old OZRs and lean Zucker rats (LZRs). Band densities were quantified by ImageJ and normalized to band density of β-actin. *A_2_*: in the medulla, tau-pSer^396^ expression was higher in OZRs vs. LZRs, but tau-pSer^199,202^ and total tau were lower in OZRs vs. LZRs. *B_2_*: in the cortex, tau-pSer^396^ expression was higher in OZRs, but tau-pSer^199,202^ expression was comparable in OZRs and LZRs, with no change in relative expression of total tau. *C_2_*: in the hippocampus, tau-pSer^396^, tau-pSer^199,202^, and total tau were higher in OZRs vs. LZRs. Data are shown as means ± SE. Group sizes were 6 or 7 rats for all comparisons. *P < 0.05, unpaired *t* test.

At 28–29 wk of age, older adult OZRs weighed ∼203 g more than the age-matched LZRs (691.6 ± 42.5 vs. 488.6 ± 19.0 g; *n* = 7/group, *P* < 0.001, unpaired *t* test). By this age, the profiles for ptau expression in OZRs and LZRs changed in the medulla, cerebral cortex, and hippocampus. In the medulla and cortex, tau-pSer^396^ expression was still higher in older adult OZRs, but expressions of tau-pSer^199,202^ and total tau were now either lower in OZRs compared with LZRs (medulla; Fig. 6*A*) or not different between OZRs and LZRs (cortex; Fig. 6*B*). In contrast to the absence of differences in the hippocampus of young adult OZRs and LZRs (Fig. 1*C*), the expressions of tau-pSer^199,202^, tau-pSer^396^, and total tau protein were significantly higher in the hippocampus of the older adult OZRs compared with LZRs (Fig. 6*C*), as previously reported for tau-pSer^396^ in adult OZRs (72). These data show a delayed development of increased ptau expression in the hippocampus in OZRs compared with earlier development in the medulla and cortex of young adult OZRs (Figs. 1 and 6). Elevated ptau in the hippocampus coincided with reductions in ptau and total tau expression in the medulla and cortex, consistent with the onset of neuronal loss previously reported in the cerebral cortex of 20-wk-old adult male OZRs (73).

#### Emergence of deficits in spatial learning and memory in older adult OZRs.

**Figure 7.**
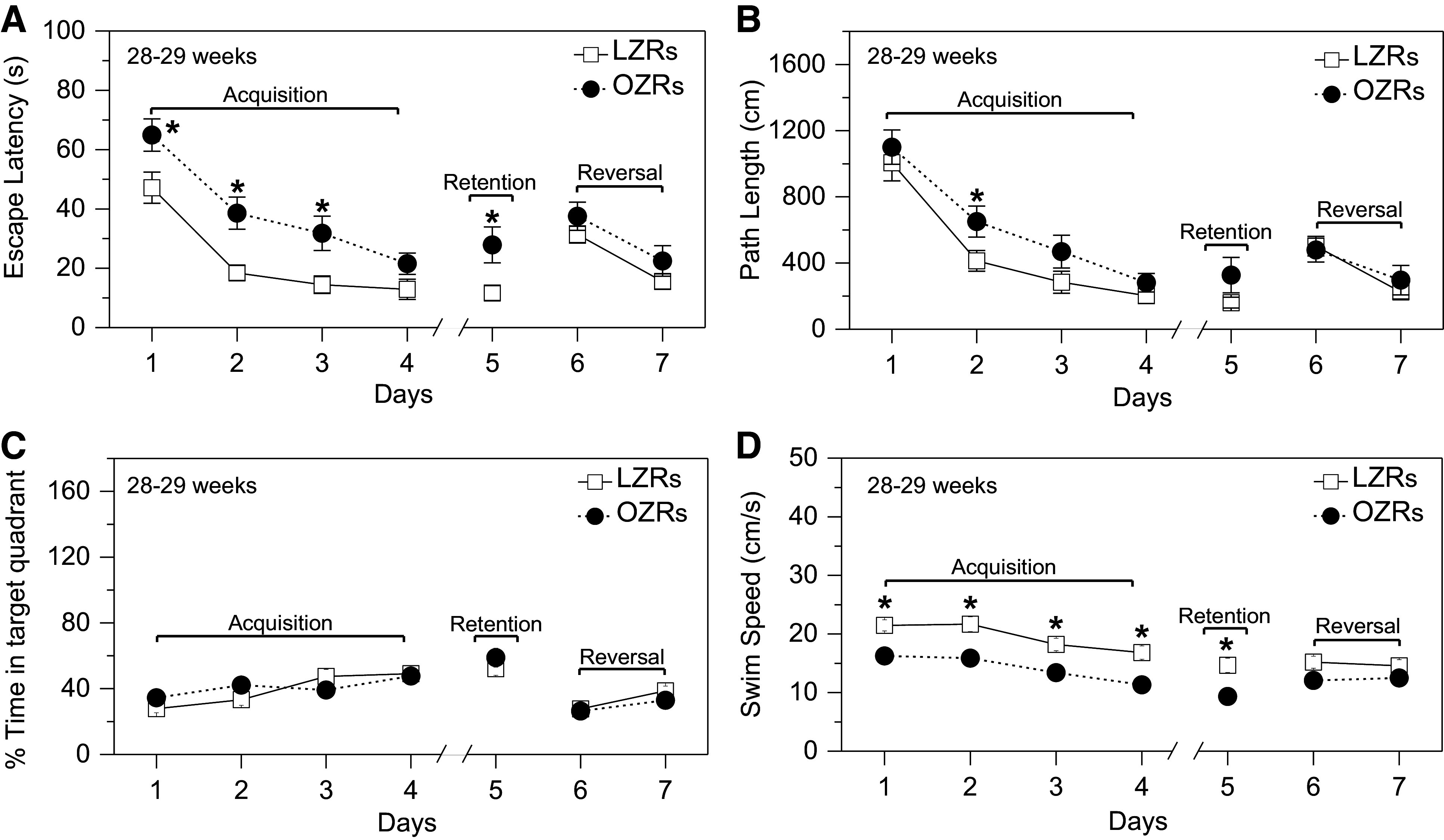
Emergence of deficits in spatial learning and memory assessed with the Morris water maze (MWM) in older adult obese Zucker rats (OZRs). *A*: escape latencies decreased with days in OZRs and lean Zucker rats (LZRs) and were significantly higher in OZRs vs. LZRs on *days 1–3* and *5*. *B*: path lengths decreased with days in OZRs and LZRs, with a longer path in OZRs vs. LZRs on *day 2*. *C*: percent time in target quadrant increased with days, with no differences between OZRs and LZRs. *D*: swim speed decreased on *days 1–5* and was slower in OZRs vs. LZRs on *days 1–5*. Data are shown as means ± SE. Group sizes were 15 LZRs and 16 OZRs for all comparisons. *P < 0.05, OZRs vs. LZRs on that day. *Days 1–5* [*period 1* (P1): acquisition and retention] and *days 6* and *7* [*period 2* (P2): reversal] were analyzed separately by 2-way repeated-measures (RM) ANOVA for days and phenotype, followed by Tukey’s post hoc tests to compare OZRs and LZRs by day when a significant main effect of phenotype was observed. *A*: escape latency: days (P1: *F* = 30.44, *P* < 0.001 and P2: *F* = 23.19, *P* < 0.001) and phenotype (P1: *F* = 15.00, *P* < 0.001 and P2: *F* = 1.87, *P* = 0.182). *B*: path length: days (P1: *F* = 39.50, *P* < 0.001 and P2: *F* = 16.06, *P* < 0.001) and phenotype (P1: *F* = 4.247, *P* = 0.048 and P2: *F* = 0.010, *P* = 0.759). *C*: percent time in target quadrant: days (P1: *F* = 13.33, *P* < 0.001 and P2: *F* = 9.20, *P* = 0.005) and phenotype (P1: *F* = 0.71, *P* = 0.405 and P2: *F* = 1.39, *P* = 0.247). *D*: swim speed: days (P1: *F* = 24.19, *P* < 0.001 and P2: *F* = 0.07, *P* = 0.800) and phenotype (P1: *F* = 32.69, *P* < 0.001 and P2: *F* = 4.17, *P* = 0.51).

**Figure 8.**
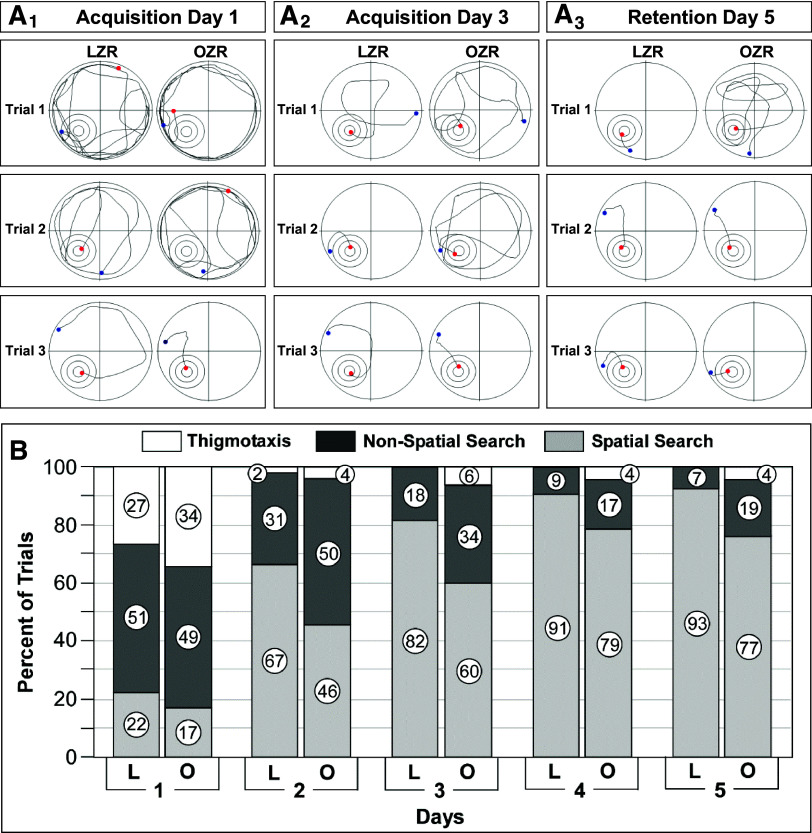
Emergence of decreased use of spatial search strategies during the Morris water maze (MWM) in older adult obese Zucker rats (OZRs). *A*: representative swim paths from 3 trials from a lean Zucker rat (LZR) and an OZR from Fig. 7 on *day 1* (*A_1_*), *day 3* (*A_2_*), and *day 5* (*A_3_*). *B:* incidence of search strategies utilized on *days 1–5* displayed as % of total trials in LZRs and OZRs from Fig. 7. Thigmotaxis was present on *days 1* and *2* but absent on *days 3–5* in older adult LZRs, but thigmotaxis persisted for *days 1–5* in some older adult OZRs. Undirected search patterns were used most often in OZRs and LZRs on *day 1*. The use of spatial search strategies increased with days as the use of nonspatial search strategies decreased, and the LZRs used spatial search strategies significantly more often than OZRs on *days 1–5*. Data analyzed by generalized estimating equations (GEEs) and Wald chi-square (54) showed a significant effect for days (χ^2^ = 23.353, *P* < 0.001) and phenotype (χ^2^ = 30.180, *P* < 0.001).

The OZRs (*n* = 16) and LZRs (*n* = 15) were 28 wk old at the beginning of the pretraining phase, and the OZRs weighed ∼216 g more than the LZRs (658.1 ± 41.7 vs. 442.6 ± 13.5 g, *P* < 0.001, unpaired *t* test). In the pretraining phase, older adult OZRs had a pronounced increase in escape latency in the morning session that was greater than age-matched LZRs and OZRs of the two younger age ranges. However, in the afternoon session the older adult OZRs improved, to yield no differences between OZRs and LZRs, as observed in the young adults (Supplemental Fig. S2). Coincident with the development of elevated ptau in the hippocampus of older adult OZRs, deficits in MWM performance also emerged in OZRs of this age range. During the acquisition phase, escape latencies decreased over 4 days in these OZRs and LZRs, but the OZRs displayed significantly increased escape latencies compared with LZRs on acquisition phase *days 1–3* and in the retention phase on *day 5* (Fig. 7*A*). This difference was due to an age-related increase in escape latencies in the older adult OZRs that was not present in the LZRs (Supplemental Fig. S4, A and B). For example, on *day 1*, escape latencies for LZRs at 28 and 18 wk of age were comparable (47.2 ± 5.2 vs. 48.7 ± 5.9 s; Supplemental Fig. S4A), but escape latencies for the OZRs were significantly increased at 28 wk compared to 18 wk (64.9 ± 5.5 vs. 48.8 ± 5.1 s; Supplemental Fig. S4B). In the older adult OZRs, a slower swim speed persisted over *days 1–5*, but the path length was only different on *day 2*, with OZRs taking slightly longer paths (Fig. 7, *B* and *D*). However, similar differences in swim speed in the young adult OZRs and LZRs on *days 1* and *2* did not yield differences in escape latencies on those days (Fig. 4*D* vs. Fig. 7*D*), in agreement with a previous finding showing that small differences in swim speed do not reliably correspond with changes in escape latencies (43). The body weights of the young adult and older adult OZRs were equivalent (655.0 ± 9.2 vs. 658.1 ± 41.7 g, respectively, *P* > 0.05, unpaired *t* test), suggesting that body weight was not a factor in the persistent reduction of swim speed or increased escape latency observed in the older adult OZRs compared with young adult OZRs (Supplemental Fig. S4B). The use of spatial search strategies increased over the 5 days in the older adult OZRs and LZRs (Fig. 8), as observed in the young adult OZRs and LZRs (Fig. 5). However, in contrast to equivalent use of spatial search strategies in young adult OZRs and LZRs, the older adult LZRs used spatial search strategies significantly more often than older adult OZRs (Fig. 8*B*). In addition, thigmotaxis persisted throughout the 5 days in some of the older adult OZRs, which was not present after 2 days in the younger adult OZRs or in the LZRs of either age range (Fig. 5*B* vs. Fig. 8*B*). Although a direct comparison of adult LZRs and OZRs by age over *days 1–5* showed no significant differences between young adults and older adults by phenotype, the LZRs trended for more use of spatial search strategies with age whereas OZRs trended for reduced use of spatial search strategies with age (Supplemental Fig. S5, C and D). Together, these data suggest that, despite similar body weights, older adult OZRs develop increased escape latencies compared with young adult OZRs, which may result from the reduced use of spatial search strategies and slower swim speed. These changes also coincide with increased ptau in the hippocampus of the older adult OZRs.

A probe test performed on *day 4* showed no differences between older adult LZRs and OZRs in the percent time spent in the target quadrant (38.4 ± 3.1% vs. 51.2 ± 5.7%) or the annulus around the target at 40 cm (23.6 ± 2.7% vs. 29.5 ± 5.3%) or 20 cm (12.3 ± 2.0% vs. 16.5 ± 3.3%), indicating that reference memory was not impaired in the OZRs during acquisition. In the reversal phase on *days 6* and *7* the escape latencies, path lengths, swim speeds, and percent time in the target quadrant were comparable in LZRs and OZRs (Fig. 7), suggesting retention of functional cognitive flexibility in the older adult OZRs (59, 68–70). After reversal testing, a second probe test showed no differences between LZRs and OZRs in the percent time spent in the target quadrant (44.4 ± 4.1% vs. 37.8 ± 4.0%) or percent time in the annulus around the target at 40 cm (34.0 ± 4.0% vs. 29.3.5 ± 3.5%) or 20 cm (19.4 ± 2.5% vs. 17.9 ± 2.6%), suggesting similar competencies in ability of the young adult male LZRs and OZRs to learn and remember a new location for the platform. Together, these data show that older adult OZRs displayed significant deficits during pretraining, which uses hippocampus-independent nonspatial learning of the task of turning around and swimming to escape the water by climbing onto a hidden platform. In addition, deficits in hippocampus-dependent spatial learning and memory during the acquisition and retention phases emerged in older adult OZRs, with more use of nonspatial search strategies, reduced swim speed, and increased escape latencies compared with older adult LZRs. However, the OZRs showed no deficits in reference memory or their ability to learn a new platform location at any of the ages.

### Adult Male OZRs Develop Changes in Phosphorylation in the Insulin/PI3K/Akt Signaling Pathway in the Cortex and Hippocampus but Not Medulla

**Figure 9.**
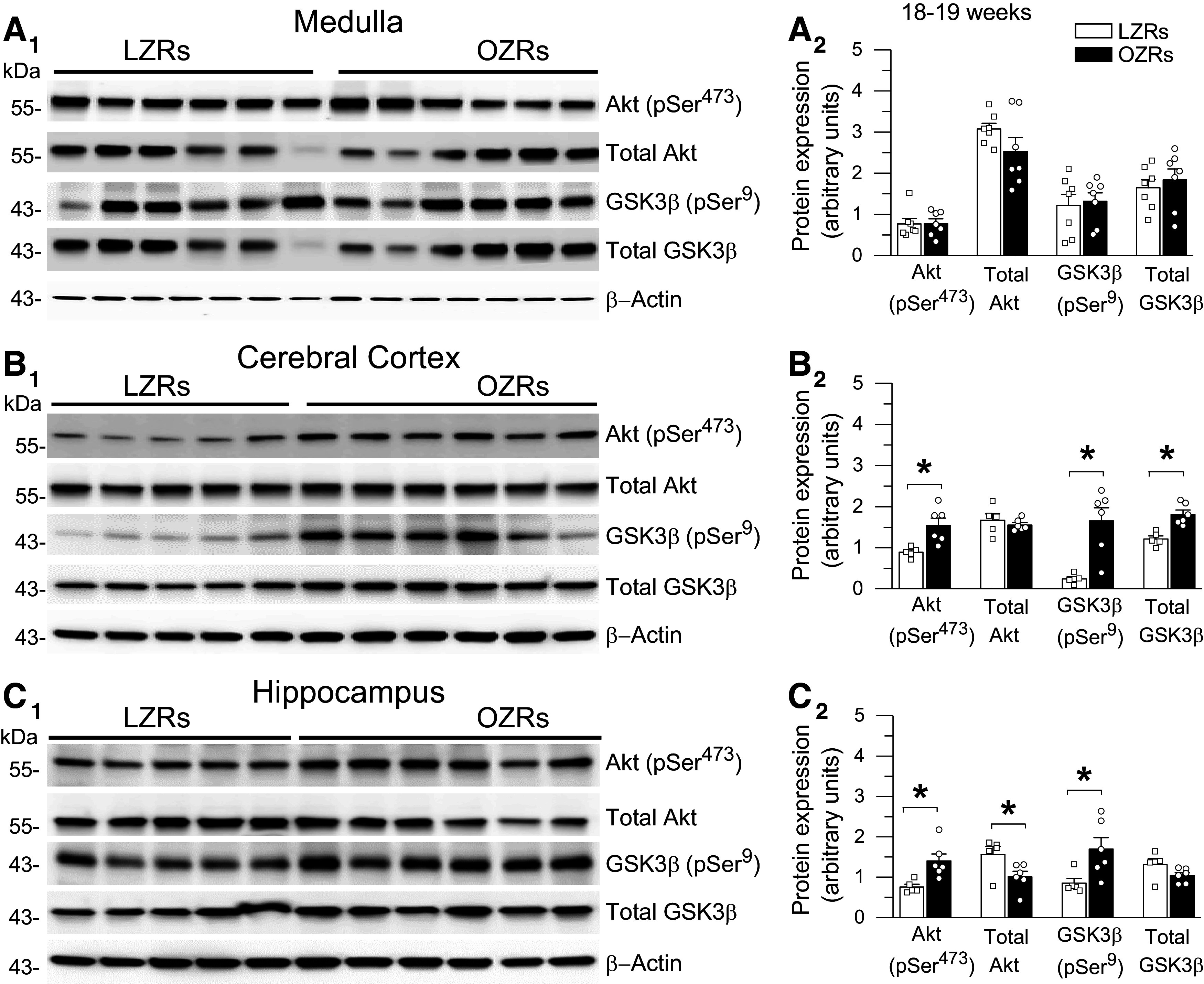
Elevated protein expression of Akt-pSer^473^ and glycogen synthase kinase-3β (GSK3β)-pSer^9^ in cerebral cortex and hippocampus but not medulla in young adult obese Zucker rats (OZRs). *A_1_* and *A_2_*: immunoblots from the medulla showed comparable levels of Akt-pSer^473^, total Akt, GSK3β-pSer^9^, and total GSK3β protein expression in OZRs and lean Zucker rats (LZRs). *B_1_* and *B_2_*: immunoblots from cerebral cortex showed higher expression of Akt-pSer^473^ and GSK3β-pSer^9^ in OZRs vs. LZRs. Total GSK3β expression was also higher in OZRs vs. LZRs, but total Akt expression was comparable in OZRs and LZRs. *C_1_* and *C_2_*: immunoblots from the hippocampus showed higher expression of Akt-pSer^473^ with lower expression of total Akt in OZRs and higher GSK3β-pSer^9^ in OZRs with comparable expression of total GSK3β in OZRs and LZRs. Data are shown as means ± SE. Rats were 130–132 days old, and group sizes were 5–7 rats for all comparisons. *P < 0.05, unpaired *t* test. See Supplemental Fig. S5 for comparisons of phosphorylated Akt and GSK3β normalized to total protein expression for these rats.

**Figure 10.**
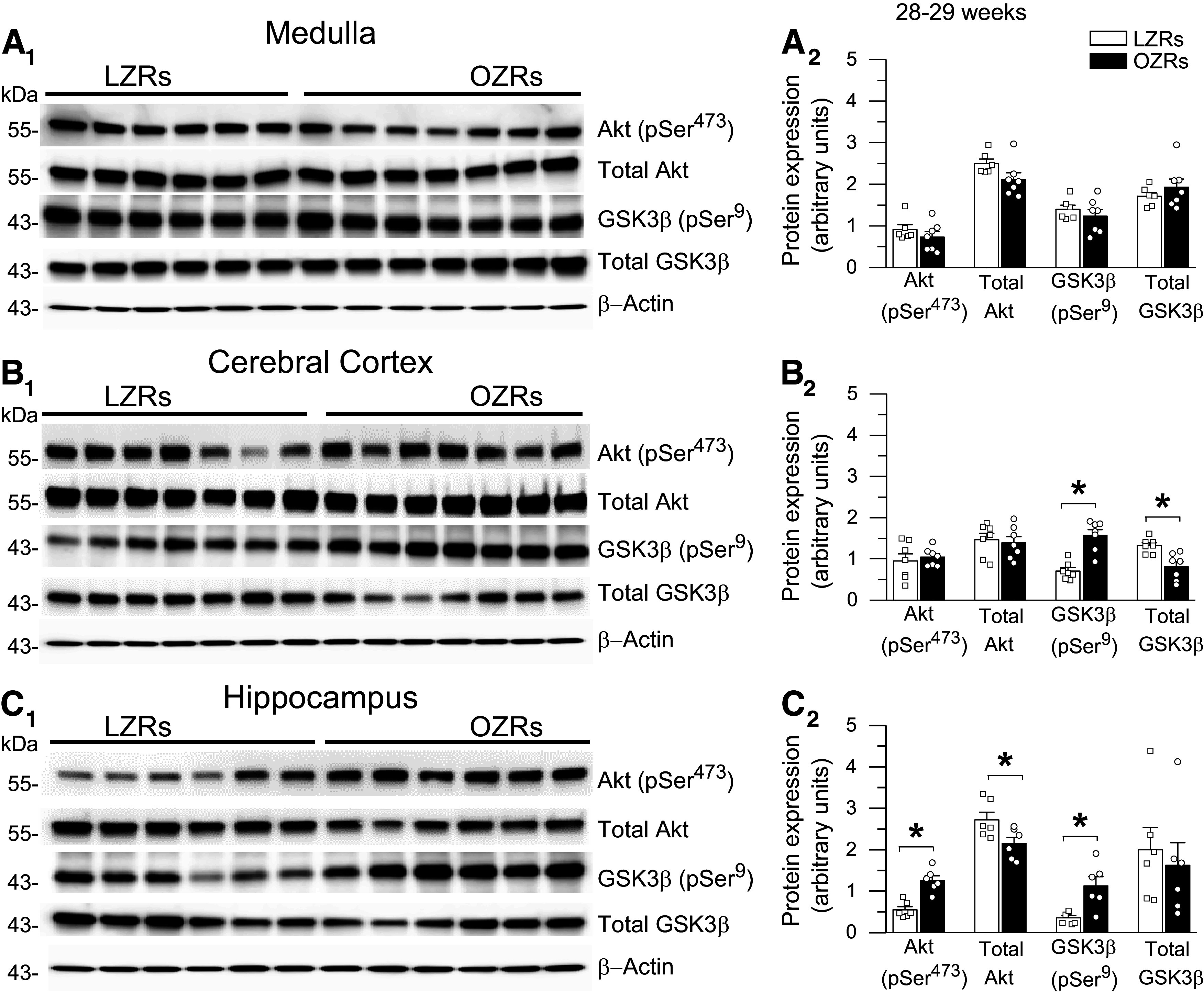
Elevated protein expression of glycogen synthase kinase-3β (GSK3β)-pSer^9^ in cerebral cortex and hippocampus but not in medulla in older adult obese Zucker rats (OZRs). *A_1_* and *A_2_*: immunoblots from medulla showed no differences in relative expression of Akt-pSer^473^, total Akt, GSK3β-pSer^9^, or total GSK3β between OZRs vs. lean Zucker rats (LZRs). *B_1_,* and *B_2_*: immunoblots from cortex showed that relative expression of pGSK3β-Ser^9^ was still higher in OZRs vs. LZRs, but total pGSK3β-Ser^9^ was now lower in OZRs vs. LZRs. Expression of pAkt-Ser^473^ was no longer different in OZRs vs. LZRs, and total Akt levels remained comparable in OZRs vs. LZRs. *C_1_,* and *C_2_*: in hippocampus, relative expressions of pAkt-Ser^473^, total Akt, pGSK3β-Ser^9^, and total GSK3β in OZRs vs. LZRs resembled those observed in young adults (Fig. 9*C*). Expression of pAkt-Ser^473^ and pGSK3β-Ser^9^ were elevated in OZRs vs. LZRs along with lower total Akt expression in OZRs and comparable total GSK3β expression in OZRs and LZRs. Data are expressed as means ± SE. Rats 28–29 wk old and group sizes of 6 or 7 for all comparisons. *P < 0.05, unpaired *t* tests.

Under physiological conditions, insulin modulates activity in the PI3K/Akt pathway by promoting phosphorylation of Akt at residue Ser^473^ (Akt-pSer^473^) to phosphorylate GSK3β at residue Ser^9^ (GSK3β-pSer^9^), which reduces GSK3β activity and its phosphorylation of tau (15, 19, 26, 27). However, in states of deficient insulin signaling, GSK3β is thought to be pathologically activated to promote hyperphosphorylation of tau (15, 19, 26, 27). In the cortex of 18- to 19-wk-old OZRs, the expressions of Akt-pSer^473^, GSK3β-pSer^9^, and total GSK3β were elevated coincident with increased tau-pSer^199,202^ and tau-pSer^396^ (Fig. 1*B* and Fig. 9*B*). At 28–29 wk of age, only GSK3β-pSer^9^ and tau-pSer^396^ remained elevated in the cortex of OZRs (Fig. 6*B* and Fig. 10*B*). In the hippocampus of 18- to 19-wk-old OZRs, elevated expression of Akt-pSer^473^ and GSK3β-pSer^9^ preceded rises in ptau and total tau (Fig. 1*C* and Fig. 9*C*). In 28- to 29-wk-old OZRs, Akt-pSer^473^ and GSKβ-pSer^9^ remained elevated in the hippocampus with the emergence of elevated tau-pSer^199,202^, tau-pSer^396^, and total tau (Fig. 6*C* and Fig. 10*C*). Examination of the relative expression in OZRs and LZRs using ratios of Akt-pSer^473^ to total Akt and GSK3β-pSer^9^ to total GSK3β yielded the same conclusions regarding differences or absence of differences in phosphorylation of these enzymes in the OZRs within the hippocampus and cortex at both adult age ranges (Supplemental Fig. S5), due to either an absence in differences in total protein expression between OZRs and LZRs or changes in phosphorylation that exceed changes in total enzyme expression (Supplemental Fig. S5).

In contrast, when relative ptau expression was rising and falling in the medulla over three age ranges in OZRs (Fig. 1*A* and Fig. 6*A*), no differences in the expression of phosphorylated or total Akt or GSK3β between age-matched adult OZRs and LZRs were observed (Fig. 9*A* and Fig. 10*A*). Together these data suggest that changes in the insulin/PI3K/Akt pathway and phosphorylation of GSK3β are indicative of insulin resistance and hyperglycemia in adult OZRs (27, 74), and changes in this pathway occurred in adult OZRs before and during increased ptau expression in the hippocampus and cortex, respectively. However, rises in ptau in the medulla of adult OZRs do not coincide with changes in phosphorylated or total Akt or GSK3β, suggesting that a different mechanism increases ptau in the medulla.

## DISCUSSION

Metabolic syndrome and ADRDs share common pathological traits, but the elements driving their comorbid onset and progression are not well understood. Animal models that mimic these human conditions in a predictable, time-dependent manner are invaluable for unraveling important insights into causative, contributing, and co-occurring factors to enable the development of preventative and counteractive remedies. In this study, adult male OZRs displayed age-related and region-specific emergence of increased phosphorylation of tau at multiple residues that serve as pathological markers of ADRDs, and these histological changes coincided with compromised physiological and cognitive functions associated with the affected brain regions. These changes also corresponded with recent development of deleterious physiological traits of MetS, suggesting that OZRs provide a useful model to investigate the connections between traits of MetS and the development of neuropathologies related to ADRDs.

### Sequential Region-Specific Changes in ptau in Adult Male OZRs

Tau is a microtubule-associated protein that contributes to the stability of neurons, and excess phosphorylation of tau causes detrimental changes in neuronal activity, morphology, and synaptic function with eventual neurodegeneration (24, 42, 75–77). We examined the expression of two phosphorylated epitopes located in the PHF-1 region (Ser^396^) and the proline-rich middle region (Ser^199,202^), because they are abnormally phosphorylated in humans with ADRDs (77). These two epitopes of the tau protein are targets of proline-directed kinases such as GSK3β, whose activity is modulated by changes in circulating insulin and glucose (15, 27, 74). In addition, excess phosphorylation of tau at the Ser^396^ residue is an early event in the pathogenesis of AD and other tauopathies that provides a useful marker to detect the onset of pathology (78).

Examination of age-matched LZRs and OZRs at three age ranges revealed age-related and region-specific sequences of rises and falls in the relative expression of tau-pSer^396^ and tau-pSer^199,202^ without differences in total tau expression until the latest age range. In the medulla and cortex, expression levels of tau-pSer^199,202^, tau-pSer^396^, and total tau were comparable in juvenile OZRs and LZRs, but the expression of tau-pSer^199,202^ and tau-pSer^396^ became elevated in both regions in young adult OZRs. Although elevated ptau expression in the cerebral cortex of rodents and humans with MetS and ADRDs is widespread across multiple regions and cortical layers (6, 11, 12, 18, 23, 25, 73), in the medulla we observed intense and discrete staining for tau-pSer^396^ concentrated in the dorsal vagal complex of a young adult OZR, as reported in brains of humans with AD (79). In older adult OZRs, tau-pSer^396^ expression remained elevated in the medulla and cortex, but relative expression of tau-pSer^199,202^ and total tau decreased in both regions, making tau-pSer^199,202^ and total tau expression comparable in OZRs and LZRs in the cortex and lower in OZRs than in LZRs in the medulla. These reductions are consistent with the development of neuronal loss observed in the cerebral cortex of OZRs by 20 wk of age (73).

In contrast to the medulla and cortex, in the hippocampus the expression levels of tau-pSer^396^, tau-pSer^199,202^, and total tau were not different in young adult OZRs and LZRs. However, in older adults, expressions of tau-pSer^199,202^, tau-pSer^396^, and total tau were increased in the hippocampus of OZRs compared with age-matched LZRs. In obese and diabetic rodents and humans with ADRDs, increased ptau expression has been reported throughout the hippocampus including CA1, CA3, CA4, and dentate gyrus, indicating pervasive dysfunction in this critical region of the brain (24, 25, 27). In addition, measures of ptau expression in the hippocampus of OZRs are likely to be affected by diminished neuronal structure and loss of neurons observed in these regions of the hippocampus in adult OZRs (42, 73). Together, these observations underscore the necessity of determining the time courses for the rise, progression, and reduction of neuropathological markers within distinct regions of the brain in the dynamic setting of MetS.

### Juvenile and Young Adult OZRs: MetS Traits, Medullary ptau, and Autonomic Dysfunction

Juvenile OZRs have excess weight gain with increased plasma triglycerides, cholesterol, corticosterone, and insulin but normal nonfasting blood glucose levels (45, 46). In addition, there are no differences in sympathetic nerve activity, mean AP, or short-term control of AP by baroreflexes between juvenile OZRs and LZRs (47, 50, 80). In agreement, microinjection of glutamate into the NTS produces comparable decreases in sympathetic nerve activity, heart rate, and AP in juvenile OZRs and LZRs (50). Raising AP actually produces enhanced c-Fos expression in the NTS of juvenile OZRs compared with age-matched LZRs, suggesting potential early changes in neuronal function in this region (50). Thus, although juvenile OZRs have developed some traits of MetS, they maintain normal autonomic regulation of AP with no detectable differences in ptau or total tau expression in the medulla or cerebral cortex compared with LZRs.

At MetS progresses, young adult male OZRs have further increases in body weight with increased plasma triglycerides and insulin but normal levels of fasting blood glucose and HbA1c (46, 47). However, with access to food, 12- to 14-wk-old male OZRs have persistent and significant nonfasting hyperglycemia observed when glucose is measured continuously by telemetry in undisturbed rats (46, 47). At this age, male OZRs develop elevated sympathetic nerve activity and mean AP along with impaired baroreflexes and increased variability of AP (46, 47, 50). As seen in hyperglycemic STZ-treated rats, these young adult OZRs now have diminished baroreceptor-mediated c-Fos expression in the NTS, and microinjection of glutamate into the NTS yields smaller decreases in SNA and AP in OZRs (46, 50, 65). Interestingly, treatment with metformin or pioglitazone normalizes nonfasting blood glucose in young adult male OZRs and improves baroreceptor-mediated c-Fos expression in the NTS and baroreflexes while mean AP and plasma insulin remain elevated (46), suggesting that the hyperglycemia itself plays an important role in diminished brain stem activation and baroreflexes that is distinct from mechanisms that raise mean AP. These data suggest that hyperglycemia directly or indirectly contributes to increased tau-pSer^199,202^ and tau-pSer^396^ that develop in the NTS and DMNV of young adult OZRs, and both likely contribute to the autonomic dysfunction. In a vicious cycle, hyperglycemia associated with insulin resistance appears to promote diminished activation of the NTS to impair baroreflexes, contributing to increased variability of sympathetic nerve activity and AP. In turn, rises in sympathetic nerve activity exacerbate insulin resistance that may enhance the progression of ADRDs (2, 7, 50, 61, 80). Whether increased variability of AP itself fosters the associated rise in ADRDs, they are linked by shared causes, or both warrants further investigation (7, 61). Given the earlier development of increased ptau in the medulla and the propagation properties of pathological tau from neuron to neuron, the extensive connections between the NTS and forebrain may serve as a gateway for early autonomic dysfunction to negatively impact higher-order functions (7, 25, 29, 77, 81). Whether development of ptau in the medulla contributes to the emergence of elevated tau-pSer^396^ and tau-pSer^199,202^ in the hippocampus in older adult OZRs remains to be determined (81).

### Young and Older Adult OZRs: Deficits in Nonspatial and Spatial Learning and Memory

Before the onset of elevated ptau in the hippocampus and deficits in the acquisition phase of the MWM, young adult male OZRs exhibited a deficiency in nonspatial learning during pretraining for the MWM. In the morning session, these OZRs exhibited increased escape latencies over the course of three trials that were absent in juvenile OZRs and even more pronounced in older adult OZRs (Supplemental Fig. S2). Because the rats were tasked to swim a narrow, straight alley in the absence of external cues, search strategies would not likely contribute to this pretraining deficit. During the afternoon pretraining sessions, performance improved in the adult OZRs of both age ranges and no longer differed from age-matched LZRs, suggesting no persistent sensorimotor deficits in the OZRs. This nonspatial learning deficit may be related to increased ptau observed in the cerebral cortex of young adult OZRs or motor skill-related regions such as the basal ganglia (52, 70, 73, 82). In the medulla, strong staining for tau-pSer^396^ in the inferior olivary nucleus (Fig. 2*B*), which is essential for Purkinje cell function and synaptic plasticity in the cerebellum, may indicate a cerebellar contribution to this observed motor learning deficit in young adult OZRs (71, 83). In agreement, lesion of Purkinje neurons in the cerebellum also increases escape latencies in the MWM (84), highlighting another complexity of these motor learning skills.

Older adult OZRs displayed increased escape latencies during the acquisition and retention phases of the MWM coincident with the emergence of elevated tau-pSer^199,202^ and tau-pSer^396^ in the hippocampus, which were both absent in young adult OZRs. More compelling, these older adult OZRs maintained a greater tendency to use nonspatial search strategies throughout the acquisition and retention phases, consistent with the diminished formation of cognitive maps and allocentric navigational skills that rely on the hippocampus (52, 70, 82). However, many of the performance measures in the MWM showed no differences between LZRs and OZRs, particularly during the probe trials and reversal phase. Thus, young adult OZRs displayed hippocampus-independent learning deficits during pretraining before the onset of increased ptau in the hippocampus, and the older adult OZRs appear to be in the early stages of hippocampal dysfunction with evolving deficiencies in hippocampus-dependent spatial learning and memory.

Other studies have reported the presence or absence of deficits in MWM performance in OZRs that do not align with our time courses and may be explained by differences in experimental design or protocols for the MWM. First, the absence of pretraining to learn the task likely contributed to differences observed during the acquisition phase in juvenile OZRs and *db/db* mice (31). In agreement, in STZ-treated diabetic rats increased escape latencies during the acquisition phase of the MWM were abolished by pretraining, even in the absence of pretraining deficits (29). Therefore, although juvenile OZRs showed no deficits during pretraining in our study, pretraining may have prevented our observation of apparent deficits previously reported during the acquisition phase in juvenile OZRs (31). Second, the detection of differences in performance depends upon the duration and severity of causal factors (85). The 18- to 19-wk-old OZRs had altered insulin signaling in the hippocampus consistent with the insulin resistance and moderate nonfasting hyperglycemia that are present in male OZRs by this age (Fig. 9; Ref. 46). However, increased ptau in the hippocampus and longer escape latencies in the MWM did not transpire until 28–29 wk of age. Likewise, deficits in hippocampal LTP reported in younger OZRs may precede observation of behavioral deficits in spatial learning and memory (31). In agreement, after 11 wk with severe hyperglycemia STZ-treated rats displayed impaired hippocampal LTP and spatial learning and memory with the MWM, which were not present with moderate hyperglycemia of a similar duration or severe hyperglycemia at a shorter duration (85). Third, the use of a less sensitive MWM protocol may be a factor in the reported absence of deficits in escape latencies in 26- to 28-wk-old male OZRs (43), which are also at the age of early stages of MWM deficits. Because rats show significant improvement during each day’s trials in the MWM (86), using 8 trials/day instead of the usual 3–4 trials/day markedly reduces the daily average escape latency (43) and impairs the ability to detect differences that may be present in each day’s early trials. Together, these findings highlight the importance of examining time courses for changes in behavior with the onset and progression of MetS traits and optimizing the MWM protocol to detect the development of changes in behaviors.

### Contributions of Leptin to Autonomic Regulation of AP and Spatial Learning and Memory

Leptin is an adipokine that is best known for its roles in the suppression of food intake and energy balance by actions in the basal forebrain (35, 87). Interestingly, leptin has also been shown to impact neuronal function in two brain regions particularly affected by MetS, namely the dorsal vagal complex and the hippocampus. Injection of leptin into the dorsal vagal complex reduces food intake and produces weight loss (88), and the absence of leptin’s actions in this region could contribute to the early hyperphagia and weight gain in OZRs (89). Leptin has also been proposed as a contributor to other NTS-mediated reflexes because injection of leptin into the NTS mimics the impact of disease states that are associated with elevated circulating leptin, such as exaggerated chemoreflexes with obstructive sleep apnea and impaired baroreflexes with obesity and MetS (90, 91). In the hippocampus, leptin interacts with insulin to inhibit GSK3β and enhances the actions of NMDA receptors to facilitate the generation of LTP (9, 31, 33, 39), which plays an essential role in complex spatial navigation, learning, and memory observed with the MWM (29, 32, 52). In support of a role of leptin for hippocampus-related learning and memory, several studies report that OZRs and *db/db* mice exhibit impaired spatial learning and memory with the MWM (14, 30, 31), but the use of limited age ranges does not elucidate whether deficits are present from birth or develop with the progression of MetS (14, 31, 42). Thus, although leptin is likely to contribute to NTS and hippocampal functions when present, in the absence of leptin’s actions normotensive juvenile OZRs have no deficits in NTS-mediated reflexes or impairments in spatial or nonspatial learning and memory with the MWM. Instead, development of deleterious traits of MetS likely underlies the emergence of these behavioral deficits in adult OZRs and other models of MetS with functional leptin receptors (14, 27, 29, 46, 47, 50, 85, 86).

### Region-Specific Changes in the Insulin/PI3K/Akt Insulin Pathway in Adult OZRs

Phosphorylation at epitopes in the PHF-1 region (Ser^396^) and the proline-rich middle region (Ser^199,202^) of the tau protein within the medulla, cortex, and hippocampus is increased in adult OZRs, other rodent models of MetS, and postmortem brains from humans with AD (18, 27, 72, 74, 78, 92). Deficient insulin signaling in the PI3K/Akt pathway that allows pathological activation of GSK3β has been proposed as a common denominator that links MetS with tau hyperphosphorylation at these epitopes (15, 19, 20, 26–28, 75, 92–96). Under normal physiological conditions, phosphorylation of GSK3β at the Tyr^216^ residue provides constitutive activation of GSK3β to promote physiological phosphorylation of tau (27, 97). Insulin acts through the PI3K/Akt pathway to activate Akt by phosphorylation at residue Ser^473^, which inhibits GSK3β by phosphorylation at residue Ser^9^ to limit phosphorylation of tau (15, 19, 20, 74, 92). Expression levels of Akt-pSer^473^ and GSK3β-pSer^9^ in the hippocampus and cortex fluctuate in positive correlation with changes in insulin that can be demonstrated by fasting to decrease glucose, insulin, and the expression of GSK3β-pSer^9^. In agreement, the injection of insulin or glucose to raise insulin rapidly increases GSK3β-pSer^9^ expression within minutes (74). Therefore, a prediabetic state that impairs insulin-mediated inhibition of GSK3β by reduced expression of GSK3β-pSer^9^ may allow pathological activation of GSK3β and tau hyperphosphorylation (27, 72, 75, 85) and contribute to the increased incidence of dementia in people with prediabetes (98, 99). However, with progression to frank diabetes, persistent hyperglycemia fosters a seemingly paradoxical rise in GSK3β-pSer^9^ in the brain. In this condition, although the ability of insulin to phosphorylate Akt is reduced or absent (27, 74, 93), acute reduction of hyperglycemia can decrease elevated Akt-pSer^473^ and GSK3β-pSer^9^ in the hippocampus within minutes (74). In our study, levels of Akt-pSer^473^ and GSK3β-pSer^9^ were higher in the hippocampus and cortex of adult OZRs compared with LZRs, which is consistent with a state of insulin resistance and the persistent nonfasting hyperglycemia observed in adult male OZRs of this age, *db/db* mice, and rats on a high-fat diet (27, 46, 74, 93). However, elevated Akt-pSer^473^ and GSK3β-pSer^9^ are not consistent with hyperphosphorylation of tau, which is also present in the cortex and hippocampus of rats with MetS (72, 74, 93).

Measurements of changes in the expression of GSK3β-pSer^9^ to infer disease-related alterations in activity have yielded conflicting results that do not necessarily reflect the activity of this kinase or its role in tau hyperphosphorylation (100). Examinations of the hippocampus in postmortem brains of humans with AD have yielded opposing findings of reduced and increased expression of Akt-pSer^473^ and GSK3β-pSer^9^ (13, 20, 94, 95, 100). Contrary to our findings in OZRs and studies reporting elevated GSK3β-pSer^9^ in the hippocampus and cortex in diabetic rats (27, 74, 93), other studies report reduced expression of Akt-pSer^473^ and GSK3β-pSer^9^ in the hippocampus of OZRs, *db/db* mice, and rats on a high-fat diet (72, 75, 92, 96). Interestingly, some of the studies reporting reduced GSK3β-pSer^9^ also stated that the rodents were fasted before euthanasia (72, 75, 92), which can normalize elevated nonfasting blood glucose in young adult OZRs (46, 72). In contrast, in our study the adult OZRs with increased GSK3β-pSer^9^ were not fasted before euthanasia, so they would be hyperglycemic at the time of death (46). These seemingly conflicting data may simply reflect the fasted or fed state at the time of euthanasia, because reducing hyperglycemia can readily lower GSK3β-pSer^9^ expression levels in rodents with diabetes (74). Although changes in expression levels of Akt-pSer^473^ and GSK3β-pSer^9^ have been shown to correlate with expected changes in their activity (74), others find that increased GSK3β-pSer^9^ occurs in a fraction of the total GSK3β to provide a relatively small inhibition of GSK3β activity or that the simultaneous phosphorylation of GSK3β at the Tyr^216^ residue overcomes inhibition by GSK3β-pSer^9^ (27, 97). These data suggest that altered levels of Akt-pSer^473^ and GSK3β-pSer^9^ reflect changes in central insulin signaling, but a snapshot measurement of GSK3β-pSer^9^ does not reflect GSK3β activity or its contributions to tau hyperphosphorylation in different stages of MetS and ADRDs (18, 74, 100).

Regardless of changes in GSK3β-pSer^9^, other evidence strongly suggests that activated GSK3β contributes to the excess phosphorylation of tau in the hippocampus of rodents and humans (19, 27, 96). Inhibition of GSK3β by systemic treatment with lithium or infusion of a non-ATP-competitive thiadiazolidinone inhibitor of GSK3β directly into the hippocampus of *db/db* mice reduces tau hyperphosphorylation and enhances hippocampal LTP (19, 96, 101). In agreement, a high-fat diet that elevates glucose and corticosterone in mice activates serum-glucocorticoid-regulated kinase 1 (SGK1) and GSK3β to promote tau hyperphosphorylation, neurodegeneration, and impaired spatial learning and memory in these mice, and SGK1 is also activated in the hippocampus of human brains with AD (27). Consistent with an insulin-resistant hyperglycemic state, these diabetic mice had elevated Akt-pSer^473^ and GSK3β-pSer^9^ along with elevated GSK3β-pTyr^216^ and increased tau-pSer^396^, suggesting a net activation of GSK3β despite elevated pGSK3β-Ser^9^ (27). However, juvenile male OZRs have elevated insulin and corticosterone by 5 wk of age, long before the onset of persistent hyperglycemia and hippocampal changes observed in adult OZRs, suggesting that hyperglycemia and its consequences are essential contributors for pathological activation of GSK3β with MetS (27, 45, 66). Together these studies suggest that in the prodromal phase of diabetes reduced activation of the PI3K/Akt cascade by deficient insulin signaling may create a period of GSK3β disinhibition that could provide insight into the association between prediabetes and increased risk of developing tau hyperphosphorylation and dementia (74, 98, 99). However, the progression into frank diabetes promotes low-grade inflammation, oxidative stress, elevated glucocorticoids, and the pathological activation of GSK3β despite elevated GSK3β-pSer^9^ that contribute to hyperphosphorylation of tau to impair cognitive functions (18, 27).

In stark contrast to the changes observed in the hippocampus and cortex, in the medulla tau-pSer^199,202^ and tau-pSer^396^ increased in adult OZRs without any changes in phosphorylated or total Akt or GSK3β. The earlier development of increased ptau in the dorsal vagal complex may reflect changes in sensory inputs and NTS neurons vulnerable to prediabetic hyperglycemia (64, 79, 81, 102–104). In agreement, normalizing nonfasting hyperglycemia in young adult OZRs restores baroreceptor-mediated c-Fos expression in the NTS and baroreflexes in young adult OZRs, suggesting that hyperglycemia is a catalyst for autonomic dysfunction by the medulla (46). Whether prediabetic hyperglycemia causes the elevated ptau in the medulla warrants further investigation. Together these data suggest a cascade of deleterious events in prediabetic young adult OZRs, whereby obesity and nonfasting hyperglycemia and its consequences increase ptau expression to impair activation of the NTS and diminish control of AP by baroreflexes, and the resulting increased variability of AP may contribute to later dysfunction in the forebrain to promote ADRDs (7–9, 46, 47, 61, 62, 79–81, 98, 102, 103).

### Perspectives

Excess phosphorylation of tau is an indicator and likely contributor to the development of autonomic and cognitive deficits observed in adult male OZRs, whose lack of functional leptin receptors drives hyperphagia-induced obesity and other traits of MetS. In young adult OZRs, expression of tau-pSer^199,202^ and tau-pSer^396^ increased in the medulla and cerebral cortex, with deficits in autonomic regulation of AP and hippocampus-independent nonspatial learning. Several months later, expression of tau-pSer^199,202^ and tau-pSer^396^ increased in the hippocampus with the onset of deficits in hippocampus-dependent spatial learning and memory. Although leptin can modulate neuronal activity in the medulla and hippocampus (90, 91), the absence of deficits in juvenile OZRs demonstrates that leptin is not required for these functions or their later impairment. Changes in insulin signaling in the PI3K/Akt pathway in the hippocampus and cortex precede and coincide with increased ptau in adult male OZRs, but in the dorsal vagal complex elevated tau-pSer^199,202^ and tau-pSer^396^ develop in the absence of changes in phosphorylated or total Akt or GSK3β by mechanisms yet to be determined. The clear development of pathology in the medulla before obvious changes in hippocampal function when young adult male OZRs have insulin resistance with nonfasting hyperglycemia but normal fasting blood glucose and HbA1c levels (46, 47) bears a disconcerting resemblance to observations in humans without diabetes whose mildly elevated blood glucose coincides with an increased risk of later dementia (98, 99). The predictable development of pathological markers with autonomic and cognitive deficits that begin when young adult male OZRs are insulin resistant and prediabetic suggests that this naturally occurring genetic model of MetS is suitable to elucidate the early driving factors of MetS that promote hyperphosphorylation of tau and the later development of ADRDs.

## DATA AVAILABILITY

Data will be made available upon reasonable request.

## SUPPLEMENTAL MATERIAL

10.6084/m9.figshare.25215557.v1 Supplemental Figs. S1–S5: https://doi.org/10.6084/m9.figshare.25215557.v1.

## GRANTS

This research was supported by the National Heart, Lung, and Blood Institute at the National Institutes of Health (Grant R01-HL-132568-S1) to A.M.S. and in part by the JES Edwards Foundation, the Summerfield G. Roberts Foundation, and a seed grant from the Department of Physiology and Anatomy at University of North Texas Health Science Center to A.M.S. and D.A.S.

## DISCLOSURES

No conflicts of interest, financial or otherwise, are declared by the authors.

## AUTHOR CONTRIBUTIONS

P.D.-E., D.A.S., and A.M.S. conceived and designed research; P.D.-E. and A.M.S. performed experiments; P.D.-E., D.A.S., N.S., and A.M.S. analyzed data; P.D.-E., D.A.S., N.S., and A.M.S. interpreted results of experiments; P.D.-E. and A.M.S. prepared figures; P.D.-E. and A.M.S. drafted manuscript; P.D.-E., D.A.S., N.S., and A.M.S. edited and revised manuscript; P.D.-E., D.A.S., N.S., and A.M.S. approved final version of manuscript.
